# Are interventions focused on gender-norms effective in preventing domestic violence against women in low and lower-middle income countries? A systematic review and meta-analysis

**DOI:** 10.1186/s12978-019-0726-5

**Published:** 2019-07-01

**Authors:** Agumasie Semahegn, Kwasi Torpey, Abubakar Manu, Nega Assefa, Gezahegn Tesfaye, Augustine Ankomah

**Affiliations:** 1 Department of Population, Family and Reproductive Health, School of Public Health, College of Health Science, University of Ghana, Legon, Accra, Ghana; 2 College of Health and Medical Sciences, Haramaya University, Po. Box 235, Harar, Ethiopia; 3 Population Council/Ghana, Yiyiwa Drive, Accra, Ghana

**Keywords:** Domestic violence against women, Systematic review, Meta-analysis

## Abstract

**Background:**

One in three women experience intimate partner violence worldwide, according to many primary studies. However, systematic review and meta-analysis of intimate partner violence is very limited. Therefore, we set to summarize the findings of existing primary studies to generate evidence for informed decisions to tackle domestic violence against women in low and lower-middle income countries.

**Methods:**

Studies were searched from main databases (Medline via PubMed, EMBASE, CINAHL, PopLine and Web of Science), Google scholar and other relevant sources using electronic and manual techniques. Published and unpublished studies written in English and conducted among women aged (15–49 years) from 1994 to 2017 were eligible. Data were extracted independently by two authors, and recorded in Microsoft Excel sheet. Heterogeneity between included studies was assessed using I^2^, and publication bias was explored using visual inspection of funnel plot. Statistical analysis was carried out to determine the pooled prevalence using Comprehensive Meta-Analysis software. In addition, sub-group analysis was carried out by study-setting and types of intimate partner violence.

**Results:**

Fifty two studies were included in the systematic review. Of these, 33 studies were included in the meta-analysis. The pooled prevalence of lifetime intimate partner violence was 55% (95% CI: 52, 59%). Of these, main categories were lifetime physical violence [39% (95% CI: 33, 45%); psychological violence [45% (95% CI: 40, 52%)] and sexual violence [20% (95% CI: 17, 23%)]. Furthermore, the pooled prevalence of current intimate partner violence was 38% (95% CI: 34, 43%). Of these, physical violence [25% (95% CI: 21, 28%)]; psychological violence [30% (95% CI: 24, 36%)] and sexual violence [7.0% (95% CI: 6.6, 7.5%)] were the pooled prevalence for the major types of intimate partner violence. In addition, concurrent intimate partner violence was 13% (95% CI: 12, 15%). Individual, relationship, community and societal level factors were associated with intimate partner violence. Traditional community gender-norm transformation, stakeholders’ engagement, women’s empowerment, intervention integration and policy/legal framework were highly recommended interventions to prevent intimate partner violence.

**Conclusion:**

Lifetime and current intimate partner violence is common and unacceptably high. Therefore, concerned bodies will need to design and implement strategies to transform traditional gender norms, engage stakeholders, empower women and integrate service to prevent violence against women.

**Protocol registration:**

PROSPERO: 2017: CRD42017079977.

**Electronic supplementary material:**

The online version of this article (10.1186/s12978-019-0726-5) contains supplementary material, which is available to authorized users.

## Plain English summary

Domestic violence against women (VAW) is a well-recognized public health concern and systematic human rights violation. It has a serious negative impact on women’s lives. Domestic VAW is common and still unacceptably high in different parts of the world as observed from several primary studies which have been conducted on the frequency and its associated factors. Additionally, some interventional studies have been conducted in some parts of the world revealed that gender-norms transformation through behavioral change and communication focused program can promote gender-equality norm and avert domestic VAW. Summarized or synthesized evidence is still needed to inform and persuade policy makers and stakeholders, so they can take an evidence based decision making approach. One of the most challenging issue is that most countries’ governments have considered VAW as a minor and socially tricky issue. There is some ambiguity as to whether VAW is a private or public matter. This systematic review and meta-analysis aimed at summarizing existing primary study findings to determine its level and associated factors, identify effective interventions to prevent domestic VAW and make key recommendations. The purpose is to contribute evidence to be used by program planners, policy makers, clinicians and other stakeholders to make an informed decision on the issue of domestic VAW. The study showed that more than half of the women experienced VAW, and almost one-third of the women have experienced current VAW. Intervention strategies should focus on traditional gender role transformation to minimize the relationship power-gap and prevent VAW.

## Background

Globally, VAW is a well-recognized public health problem and a gross pervasive violation of human rights. About 35% of women experience VAW [1, 2], and almost two-third of women murders are committed by their intimate partners every year. About five percent of the women’s total health years loss has been attributed by domestic VAW which is also exacerbated by authority inequity in relationship [3, 4]. Additionally, VAW causes ill health and its associated devastating outcome are more than the cumulative problem of cancer, road traffic accidents and malaria which are massive threat and an uncontrollable public health challenge for the upcoming generation [4]. VAW is sturdily interconnected with gender inequality that affects women’s negotiation ability about reproductive health and related issues [5–7]. The expenses associated with VAW has been estimated to be 3.7% of the countries’ gross domestic product, which is almost comparable with what several countries devote on primary education [8]. Yet, it has been considered exclusively as private matter and negligible issue by the governments’ of various countries, hence not perceived as a crime [4, 5, 9]. 

Global and regional commitment to fighting domestic VAW is reflected in various international statues. For example, the United Nations aims to build an enabling household situation in improving women’s right, their political participation, economic empowerment and safety [10]. In addition, provision of comprehensive and universal access to sexual and reproductive health care has been a strategy to avert domestic VAW in the Maputo Plan of Action (2016-2030) [11]. Further, research evidence has revealed that women empowerment and community mobilization are the most recommended interventions to minimize the expenses associated with VAW and its consequences [12]. The problem of domestic VAW is caused and exacerbated by poverty, alcohol consumption and societal receptive attitude towards inequitable gender-norms, which has been exhibited through denying access to education; lack of autonomy and justifying wife-beating by fellow women [9, 13–20].

Furthermore, women’s experience of domestic violence is significantly associated with several and multiple poor physical and mental health outcomes [1, 9, 14, 17, 21–27]. Likewise, VAW has been associated with various poor reproductive health conditions such as HIV, unintended pregnancy and unsafe abortion [1, 4, 7, 9, 15–18, 20, 25, 28–41]. Therefore, VAW needs a comprehensive approaches to empower women economically, transform traditional gender-norms in improving their communication and negotiation skills [9, 17, 42]. There is a paucity of summarized evidence on; the level of domestic VAW, its associated factors, proven evidence on the technical approach and key research recommendations in low and lower-middle income countries (LLMICs). However, many primary studies have been conducted in LLMICs. The main purpose of this systematic review and meta-analysis was to summarize existing primary studies in LLMICs to determine the prevalence of domestic VAW and its associated factors; to identify effective and proven interventions and make key recommendations. It will provide an insight to policy makers, program planners, clinicians, researchers and other stakeholders to make an informed decisions on issues related to VAW.

### Review question(s)

- What was the level of domestic VAW in LLMICs?
- What were the factors associated with domestic VAW in LLMICs?
- What were the research evidence that should translate into routine action in LLMICs?
- What were the studies’ key recommendations on the prevention of domestic VAW in LLMICs?

### Method development and protocol registration

The protocol for the systematic review and meta-analysis has been registered in the International Prospective Register of Systematic Reviews (PROSPERO) (ID: CRD42017079977). This systematic review and meta-analysis methods was written according to the preferred reporting items for systematic review and meta-analysis (PRISMA) guideline [43]. The filled PRISMA checklist is attached as Additional file 1.

### Searching methods and identifications of studies

Studies were searched using medical subject headings (MeSHs), manual and email methods. Main electronic databases [Medline via PubMed, EMBASE, CINAHL, PopLine and Web of Science], direct Google search and other relevant sources were used to access studies before December 31st, 2017. In addition, emails were sent to authors whose studies were included to request studies. In addition, relevant citations from retrieved studies were searched. Search strings were constructed using keywords and their combinations based on the review questions. However, search strings were modified to suit to the databases interface accordingly. The overall detail of the search strategies are presented as Additional file 2: 2-1 to 2-4. The overall search results were exported to the Endnote citation manager software [44], and duplicate studies were removed.

### Eligibility criteria and type of studies included

Observational and interventional studies were eligible to determine the level of, and factors associated with domestic VAW as well as effective interventions and key recommendations to prevent VAW. In addition, published and unpublished studies that have been conducted on women (15–49 years) in LLMICs to assess VAW and were written in English (1994–2017) were eligible for the systematic review. The LLMICs were selected based on the World Bank’s country classification [45]. Case series, editorials, commentaries, life stories and fact sheet reports on VAW were excluded.

### Selection of studies

**Fig. 1:**
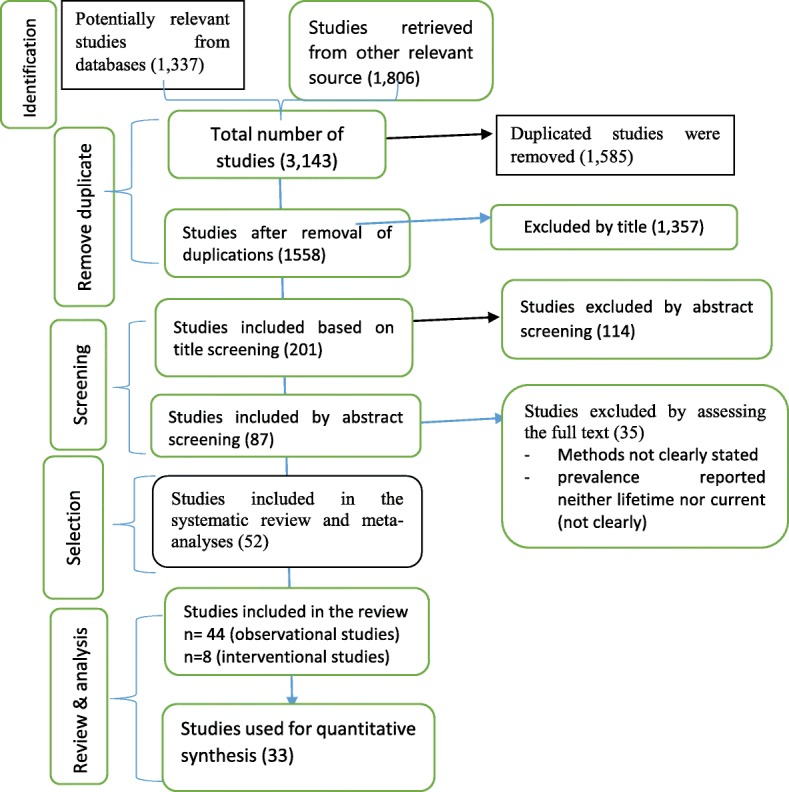
Diagramatic presentation of the selection process of studies for systeamtic review

Studies were selected using eligibility criteria and screened through four steps for the systematic review and meta-analysis. Initially, studies were screened and selected for subsequent evaluation based on their titles and abstracts, that is, if studies clearly reported on domestic VAW and its associated factors. Secondly, the two authors (AS and GT) independently screened the studies’ abstract section (aims, methods, results and conclusion) to proceed to the next step. Studies whose abstract section briefly reported the prevalence of domestic VAW and its associated factors were included in the next evaluation process. Thirdly, studies selected by abstract screening were re-assessed independently by authors (AS and GT) with focus on the full-text. Eventually, selected studies were appraised for final inclusion in the systematic review and meta-analysis. In cases where the authors could not reach a consensus on studies, a third person was involved to appraise using same checklist, in the hope of helping to make a final decision. The studies selection process adhered to the PRISMA flow diagram [43] (Fig. 1).

### Measurement of outcomes and exposures

The two phrases [domestic violence and intimate partner violence (IPV)] were interchangeably used as an outcome variable in this systematic review process. The outcome was assessed based on the prevalence of domestic/intimate partner VAW (psychological, physical, sexual and concurrent). IPV was presented in two forms (lifetime versus current). The lifetime IPV was assessed using the studies’ report of women’s experience of IPV during their lifetime. Furthermore, women’s experience of IPV over the last 12 months preceding the survey was labeled as current IPV. Exposure variables were classified according to the ecological model (individual, relationship, community and societal level factors).

### Quality assurance of the systematic review

Published and unpublished studies were searched for and considered for this systematic review to minimize publication bias. The electronic, manual and email search strategies were carried out to ensure comprehensive retrieval of studies from main databases and other relevant sources. Eligibility, quality assessment criteria, selection process and data extraction templates were properly designed by the authors to assure quality. Methodological quality assessment of the studies was carried out using the Joanna Briggs Institute (JBI) critical appraisal checklist for observational studies [46], for more detail of the critical appraisal is presented as Additional file 3. The authors (AS and GT) performed the selection of studies and data abstractions. Any disagreements were resolved through consensus, and sometimes, other authors who were not involved in data extraction adjudicated to make final decision. Potential publication biases were explored using the funnel plot. Detail of funnel plots for each outcome variables is included as Additional file 4: 4-1. Heterogeneity between included studies was assessed using I^2^. Sub-group analysis, random effects model and qualitative narration were carried out to minimize the risk of bias. In addition, the risk of bias (ROB) for interventional studies was assessed. The detail of the risk of bias assessment is attached as Additional file 4: 4-2.

### Study description and data extraction

The studies’ characteristics (authors-date, study area/country), its aims, designs, sample size, sampling procedure, response rate, key findings and recommendations are described on a template. More details of the studies characteristics description are presented on tables [see Tables 1 and 2]. Two authors (AS and GT) abstracted the data from selected studies and labeled the data extraction template using Microsoft Excel sheet. Quantitative data, number of women who had experienced VAW (labeled = Yes), who had not experienced VAW (labeled = No) and total participants (n) were separately recorded in a Microsoft Excel sheet. The more detail of abstracted data is stored in Excel Sheet (see Additional file 5).

**Table 1:** description of observational studies included in the systematic review and meta-analysis

Author, country	Study aim	Design	Population	Sampling procedure	sample	RR	Main findings	Authors key conclusion and recommendation
Sapkota et al. 2016, Nepal [47]	To estimate the magnitude of different forms of domestic violence and identify its associated factors	Cross-sectional	Married women (15–49 years)	Systematic random sampling	355	NR	The prevalence of lifetime and current physical IPV were 29.6 and 15.2%, respectively. While sexual IPV was 6.8 and 2.3%, and psychological IPV was 31.0 and 18.3%. The overall lifetime and current IPV were 38.6 and 23.1%., respectively. Furthermore, concurrent IPV was12.4%. Husband’s controlling behavior and having poor mental health were found to be at higher risk of IPV.	Domestic/ IPV is still rampant in the society with several forms. Differentials power in relationship and poor mental health was found to be positively associated with violent episodes.
Fikree F. et al., 2006, Pakistan [48]	To assess the magnitude and determinants of IPV before and during pregnancy	Cross-sectional	Pregnant women (15–49 years)	Systematic sampling	300	NR	Women’s lifetime physical and sexual IPV were 44 and 36%, respectively. Women who were ever physically abused and all reported verbal abuse. Wife’s education and duration of marriage were significantly associated to violence. 55% of the women believed that antenatal care clinics were a good time to enquire about IPV.	Almost one million Pakistani women are physically abused at least once in lifetime. RH stakeholders should be encouraged to advocate for domestic violence screening
Semahegn et al., 2013. Ethiopia [49]	To determine magnitude of domestic violence and identify its predictors	Cross-sectional	married women (15–49 years)	Systematic sampling	682	100%	The prevalence of DVAW was 78.0%. Psychological, physical and sexual violence were 73.3, 58.4 and 49.1%, respectively. Husband alcohol consumption, being pregnant, low decision making power and annual income were predictors of domestic violence	Awareness creation to avoid traditional gender norm, and support wife via integrating with community health program.
Ali et al., 2014. Sudan [50]	To investigate level and factors associated with VAW	Cross-sectional	Women (15–49 years)	Multistage sampling	1009		The prevalence of physical, psychological and sexual violence was 33.5, 30.1 and 47.6%. Husband education, polygamous marriage, and alcohol consumption were significantly associate factors.	The prevalence of domestic VAW is high in eastern Sudan.
Hayati et al. 2011, Indonesia [51]	To examine associations between IPV and husbands, psychosocial, behavior, attitudes and gender roles	Longitudinal	Women (15–49 years)	Random sampling	765	NR	Lifetime exposure to sexual and physical IPV were 22 and 11%. Sexual IPV was associated with husbands’ age (less than 35 years and educated less than 9 years). Exposure to physical violence was strongly associated with husbands’ being unfaithful, using alcohol, fighting, having childhood witnessed and the attitudes and norms expressed by the women confirm that unequal gender relationships.	Women who did not support the right of women to refuse sex were more likely to experience physical IPV. Those who justified wife-beating were more likely to experience sexual IPV. Women’s risk of IPV is due to traditional gender-norms.
Doku and Asante, 2015. Ghana [52]	investigates factors that influence women approval of domestic physical violence	Longitudinal survey	Women (15–49 years)	Two stage sampling	10,607	NR	IPV was 39%. Women aged (< 34 years) were more likely to approve physical IPV than aged 35 years and above. Women with no education (OR = 3.1, CI:2.4–3.9), primary education (OR = 2.6, CI:2.1–3.3) and secondary education (OR = 1.8, CI:1.4–2.2) had higher risk to physical IPV than women who had secondary education or higher. Women belonging Muslims (OR = 1.5, CI:1.3–1.8) and traditional believer (OR = 1.7, CI:1.2–2.4) were more likely to physical VAW. Women in the richest, rich and middle wealth index were less likely to physical VAW of wives compared to the poorest.	Interventions and policies should be geared at contextualizing intimate partner violence in terms of the justification of this behavior, as this can play an important role in perpetration and victimization.
Dalal K et al., 2014, Nepal [53]	To examines the associated factors at various level of the victims of IPVAW	Cross-sectional	Women (15–49 years)	Multistage sampling	4210	NR	IPV was 32.4%. Emotional, physical and sexual IPV were 17.5, 23.4 and 14.7%, respectively. Joint decision making for contraception, husband’s non-controlling behavior and friendly feelings were emerged as less likely to be IPV.	The findings have immense policy importance as a nationally representative study and indicating necessity of more gender equality.
Sambisa W. et al., 2011 Bangladesh [54]	explored the prevalence and correlates of past-year physical VAW	a population-based survey	Women (15–49 years)	multi-stage cluster sampling	9122		The current physical IPV was 31%. The risk of physical IPV was lower among older women, women with post-primary education and belonging to rich households and women whose husband considered their opinion in decision-making. Women were at higher risk of abuse if they lived in slums, had many children and approved wife beating norms.	Physical IPV in urban Bangladesh demonstrating the seriousness of multifaceted phenomenon as a social and public health issue that needs a comprehensive intervention strategies.
Abate et al. 2016. Ethiopia [27]	To assess the prevalence and associated factors of IPV during recent pregnancy	Cross-sectional	women (15–49 years)	Simple random sampling	282	94.3	The prevalence of IPV was 44.5%. More than half (55.5%) experienced all three forms of IPV. The joint occurrence of IPV was 56.5%. Dowry payment decreases IPV (AOR 0.09, 95% CI 0.04, 0.2) and pregnant women whose marriage didn’t undergo marriage ceremony were 79% were less likely to experience IPV (AOR 0.21, 95% CI: 0.1, 0.44).	Increasing community awareness about the consequences of the practice could be important through community health workers.
Rapp et al., 2012, Bangladesh & India DHS [55]	To investigate the association between spousal education gap and domestic violence	Population based surveys (DHS)	Married women (15–49 years)	Multi stage random sampling	69,805	NR	IPV was 52.1% in Bangladesh and 69.7% in India. Wives with higher education than their husband were less likely experience violence as compared with equal or less education. Equally high educated couples raveled the lowest likelihood of experiencing domestic violence.	Further research should be done to reveal unknown determinants so that suitable interventions to reduce DV can be developed
Dhakal L et al., 2014. Nepal [14]	To examine the relationships between IPV and STIs	Cross-sectional DHS survey	Women (15–49 years)	Two stage stratified cluster sampling	3114	NR	Approximately 15% of married women experienced some form of IPV. The odds of getting STI were 1.88 [95% CI:1.29, 2.73] times higher among women exposed to any form of IPV in compared to women not exposed to any form of IPV	IPV was common issue. Integration of IPV prevention and RH programs is needed to reduce the burden of STIs.
Rahman M, 2015. Bangladeshi [38]	To assess the association between IPV and TOP among married women	Population based survey (DHS)	Married pregnant women (15–49 years)	A stratified, multistage cluster sample	1875	NR	The experience of IPV was 31.4%. The experience of sexual and physical IPV were 13.4 and 25.8%, respectively. Physical IPV was significantly associated with both TOP ever (OR = 1.36; 95% CI: 1.05–1.77) and TOP in last 5 years (OR = 1.72; 95% CI: 1.11–2.06).	Prevention of IPV which was associated with pregnancy termination may reduce the high incidence of termination of pregnancies in Bangladesh.
Tumwesigye et al. 2012 Uganda [56]	To assess the pattern and levels of PIPVAW and its associated factors	(UDHS 2006)	Women (15–49 years)	Two stage cluster systematic sampling	1743	99.7%	Physical IPV was 48%. Women whose partner got drunk often were 6 times more likely report PIPV (95% CI: 4.6, 8.3) as compared with never drunk. The higher the education level of women the less likelihood of experience of IPV.	IPV preventive measure should address reduction of drinking among men, empowerment of women via education, employment and increased income.
Yigzaw T et al., 2004. Ethiopia [25]	To assess the prevalence of domestic violence and associated factors	Cross-sectional	Women (15–49 years)	Systematic sampling	1104	NR	IPV was 50.8%. Physical violence was found to be 32.2%, while that of forced sex and physical intimidation amounted to 19.2 and 35.7%, respectively. Exposure to parental violence as a girl was the strongest risk factor for being victim of violence later in life while alcohol consumption was the major attribute of IPV.	IPV is highly prevalent. Its prevention should be comprehensive and multi-faceted. Women prefer educational approach to minimize IPV through IEC, empowerment and legal reform.
Delamou et al., 2015, Guinea [57]	To describe the prevalence and correlates of IPV Family Planning users	cross-sectional study	Women (15–49 years)	All women who attend the clinic	232	NR	Lifetime, IPV was 92%. Where, psychological, sexual and physical IPV were 79.3, 68.1 and 48.4%, respectively. Joint occurrence IPV was 24%. IPV was higher in women with secondary level of education than higher level of education (AOR: 8.4; 95% CI 1.2–58.5).	A holistic approach that includes promotion of women’s rights and gender equality, existence of laws and policies is needed to prevent and respond to IPV.
Kabir Z et al., 2014 Bangladeshi [58]	To investigate the association between IPV and maternal depression	Longitudinal study	Women (15–49 years)	Convenient	660	NR	Prevalence of physical, sexual and emotional IPV were 52, 65 and 84%, respectively. The husband’s education (OR: 0.41, CI: 0.230.73) and a poor relationship with the husband (OR: 2.64, CI: 1.076.54) were significantly associated with IPVAW.	It is important to screen for both IPV and depressive symptoms during pregnancy and postpartum.
Kazaura et al., 2016. Tanzania [59]	To determine the magnitude of IPV and associated factors	Cross section	Women (15–49 years)	Systematic sampling	471	NR	The lifetime IPV was 65% with 34, 18 and 21% reporting current emotional, physical and sexual violence, respectively. The prevalence of women perpetration to physical IPV was above 10% regardless to their exposure to emotional, physical or sexual IPV.	IPV towards women was high. Based on hypothesis of IPV and HIV co-existence, there should be strategies to address the problem of IPV especially among women
Kouyoumdjian et al.2013, Uganda [60]	To identify risk factors for IPV in women of the reproductive age in Rakai district of Uganda	Rakai community Cohort (2000–2009)	Women (15–49 years)	Cluster sampling	15,081	NR	Lifetime and current IPV were 49.8 and 29.0%, respectively. The risk of IPV associated with sexual abuse during young age, early age of first sex, lower level of education, forced first sex, relationship of short duration, having partner of same age or younger, alcohol use and thinking that violence is acceptable.	These findings are useful for the development of prevention strategies to prevent and mitigate IPV in women.
Rahman et al. 2012, Bangladeshi [41]	To explore the association between IPV and use of RH care	DHS, 2007	Married women (15–49 years)	multi-stage cluster sampling	2001	NR	Physical IPV was 48%. Sexual IPV violence was 18.7, and 14.1% was experienced both physical and sexual IPV. Maternal experience of IPV was associated with low use of receiving sufficient ANC.	There is an association between exposure to IPV and lower use of reproductive health care services
Deyessa N. et al., 2010 Ethiopia [61]	To explore VAW in a low-income setting	Cross-sectional	Women (15–49 years)	simple random sampling	1994	NR	Women had beliefs and norms favoring VAW, living in rural and illiterate women were more likely to experience VAW. Literate rural women who were married to an illiterate spouse had the highest odds of IPV (AOR, 3.4; 95% CI: 1.76.9).	Semi-urban lifestyle and literacy promote changes in attitudes and norms against IPV.
Karamagi et al., 2006. Uganda [62]	To determine prevalence of IPV and identify risk factors	Cross-sectional	Women (15–49 years)	Cluster survey method	457	NR	The life time and current IPV were 54 and 14%, respectively. Women having higher education and satisfied marriage were associated with low risk of IPV, while alcohol consumption, rural residence and husband having multiple sexual partner were associated with high risk of IP.	IPV is linked with gender inequality, alcohol, poverty and multiple sexual partner. Programs for the prevention of IPV need to target these underlying factors.
Das et al.2013 India [63]	To describe the level of IPV and its social determinants	Cross sectional	Women (15–49 years		2139	NR	The prevalence of IPV was 15% in which physical, sexual and psychological IPV were 12, 2 and 8%, respectively. Almost one- third (35%) of IPV was justifiable. The experience of IPV was associated with poorer families and husband alcohol use.	The element of violence are mutually reinforcing and need to be taken into account collectively and framing public health initiatives.
Burgos-Soto J. et al., 2014. Togo [35]	To describe the effect of IPV on care-seeking behaviors of women	Cross-sectional	Women (15–49 years)	Systematic sampling	454	NR	Lifetime physical and sexual IPV among HIV-infected women were significantly higher than among uninfected women (63.1 vs. 39.3% and 69.7 vs. 35.3%). IPV was strongly associated with male partner multi-partnership, early start of sexual life and gender submissive attitudes.	IPV screening should be carried out at health-care settings. Couple-oriented HIV prevention interventions and couple dynamics in terms of IPV is needed.
Yimer T. et al., 2014. Ethiopia [64]	To assess the magnitude of domestic violence and its associated factors among pregnant women	Cross-sectional	Women (15–49 years)	multistage sampling	425	97.9%	IPV was 32.2%. Psychological, sexual, and physical IPV were 24.9, 14.8, and 11.3%, respectively. Married women (≤15 years) (AOR, 4.2,95%CI;1.9–9.0); childhood witness (AOR = 2.3,95%CI;1.1–4.8), having drinker partner (AOR = 3.4, 95% CI 1.6–7.4), and undesired pregnancy by partner (AOR = 6.2, 95% CI 3.2–12.1) were the main significant factors.	Domestic violence during current pregnancy is high which may lead to a serious health consequence both on the mothers and on their fetus.
Dalal K et al., 2013. Bangladeshi [65]	to examine the associations between microfinance programme membership and IPV	Cross-sectional	Married women (15–49 years).		4465	NR	Physical IPV was 48%. For women with secondary or higher education, and women at the two wealthiest levels of the wealth index, microfinance programmes membership increased the exposure to IPV. Educated women who were more equal with their spouses in their family relationships in decision-making increased their exposure to IPV.	Microfinance plans are associated with an increased exposure to IPV among educated and empowered women.
Eme T Owoaj et al., 2012, Nigeria [66]	To determine the prevalence of physical violence and the factors predisposing women in a low-income community	Cross-sectional	Women (15–49 years)	cluster sampling	924	98.6%	The prevalence of lifetime experience of physical IPV was 28.2%. The significant predictors for physical IPV were previous experience of psychological abuse (aOR: 4.71; 95% CI: 3.23–6.85); sexual abuse (aOR: 5.18; 3.21–8.36); having attitudes supportive of IPV (aOR: 1.75; 1.2–2.4); partner’s daily alcohol consumption (aOR: 2.85; 1.50–5.41); and previous engagement in a physical fight (aOR: 3.49; 1.87–6.50).	Community based IPV prevention programmes targeted at breaking the cycle of abuse, transforming gender norms which support IPV and reducing alcohol consumption should be developed
Laisser et al. 2011. Tanzania [67]	To explore community members’ understanding and their responses to IPV.	Ground theory/qualitative study	Community members	Purposive sampling	75	NR	Moving from frustration to inquiring traditional gender norms that denoted a community in transition where the effects of IPV had started to fuel a wish for change. Justified as part of male prestige illustrates how masculinity prevails to justify violence. Results in “emotional entrapment” shows the shame and self-blame that is often the result of a violent relationship.	Raising of the human rights perspective, as well as actively engaging men, re-enforcement of legal rights, and provision of adequate medical and social welfare services.
Deribe K et al., 2012 (Ethiopia) [68]	to assess the magnitude of IPV in Southwest Ethiopia in predominantly rural community	Cross-sectional	Women (15–49 years)	Systematic sampling	845	100%	The lifetime prevalence of sexual or physical IPV, or both was 64.7%. The lifetime sexual and physical violence were 50.1 and 41.1%, respectively. 41.5% of women experienced physical and sexual IPV concurrently, in the past year. Men who were controlling were more likely to be violent against their partner.	Physical and sexual VAW is common. Interventions targeting controlling men might help in reducing IPV.
Antai and Adaji, 2012. Nigeria [40]	To examine the role of community-level norms and association between IPV and TOP	cross-sectional study	Women (15-49 years)	Multistage cluster sampling	19,226		IPV was 22% (physical, sexual and emotional IPV were 15, 3 and 14%, respectively). IPV types were significantly associated with factors reflecting relationship control, relationship inequalities, and educational level, justified wife beating, age of first marriage, and contraceptive use.	Further research recommended on IPV screening on pregnancy terminated site.
Kapiga et al.2017 Tanzania [69]	known about the prevalence of this type of behavior and other related abuses in Tanzania	Cross sectional (baseline for RESPECT RCT study)	Women (15–49 years	Random sampling	1021	97.3%	Lifetime and current IPV were 61 and 27%, respectively. Lifetime economic abuse and current emotional abuse were 34 and 39%, respectively. Age and socio-economic status, physical violence (OR = 1.8; 95% CI: 1.3–2.7) and sexual violence (OR = 2.8; 95% CI: 1.9–4.1) were associated with increased poor mental health.	The high prevalence of IPV and its strong links with symptoms of poor mental health underline the urgent need for developing and testing appropriate interventions to tackle both IPV and abusive behaviors.
Feseha et al.2012. Ethiopia [70]	to assess the magnitude of intimate partner physical violence and associated factors.	Cross-sectional	Women (15–49 years)	Simple random sampling	422	100%	The current physical IPV and lifetime were 25.5 and 31.0%, respectively. Significant risk factors associated with experiencing physical IPV were being a farmer (AOR, 3.0, 95%CI: 1.7, 5.5), knowing women in neighborhood whose husband to beat them (AOR, 1.87, 95%CI: 1.0, 3.5), Muslim (AOR, 2.4, 95%C.I: 1.107, 5.5), and having a drunkard partner (AOR = 2.1, 95%C.I:1.0, 4.5).	Physical IPV is serious problem among women. Multifaceted interventions such as male counseling, increasing awareness on the consequences of IPV and the effect of substance use like alcohol will help to reduce IPV.
Osinde et al., 2011. Uganda [71]	To assess the prevalence and factors associated with IPV among HIV infected women attending HIV care in Kabale Hospital, Uganda.	Cross-sectional	Women (15–49 years)	Simple SRS	317	NR	The prevalence of lifetime and current IPV were 36.6 and 29.3%, respectively. The prevalence physical and sexual were 17.6 and 12.1%, respectively. There was a significant but inverse association between education level and physical IPV (ARR, 0.50, 95% CI: 0.31–0.82), and sexual/psychological IPV (ARR, 0.47; 95%CI: 0.25–0.87). Likewise, there was a significant inverse association between education level of the spouse and IPV (ARR, 0.57, 95% CI 0.25–0.90). Use of ART was associated with any type of IPV (ARR 3.0. 95%CI 1.2–8.5).	Most of HIV positive women experienced IPV. Likewise, women who were taking antiretroviral drugs for HIV treatment were more likely to report any type of IPV. The implication of these findings is that women living with HIV especially those on antiretroviral drugs should be routinely screened for IPV.
Yigzaw T et al. 2010. Ethiopia [72]	To assess community perceptions and attitude towards violence against women by their spouses Methods	Qualitative	Key informant	Purposive	46	NR	The normative expectation that conflicts are inevitable in marriage makes it difficult for society to reject violence. Acts of VAW represent unacceptable behavior according to existing social and gender norms when there is no justification for the act and the act causes severe harm. There is considerable permissiveness of violent acts. Marital rape is not understood well and there is less willingness to condemn it.	There is insufficient understanding of VAW and many people hold a non-disapproving stance regarding violence against women by their spouses calling for a culturally sensitive information, education and communication intervention.
Uthman OA, et al., 2011. Nigeria [73]	To develop and test a model of individual- and community-level factors of IPV	Cross-sectional study (NDHS 2008)	Women (15–49 years)	Stratified multistage cluster sampling	8731	NR	Physical, sexual and emotional IPV were 10.4, 2.3 and 14.3%, respectively. Childhood witnessed, tolerant attitudes towards IPV and women with tolerant attitudes and community with tolerant attitudes were more likely to have reported IPV.	Public health interventions designed to reduce IPVAW must address people and the communities’ tolerant attitude in which they live in order to be successful.
Bamiwuye and Odimegwu, 2014, 6 SSA countries [74]	To examine whether women from poor households are more likely to experience violence from husband than other women who are from middle or rich households.	Cross-sectional studies (DHSs)	Women (15–49 years)	Multistage cluster samplings	38,426	NR	The six SSA countries IPV was 40.5%. Physical, sexual or emotional) ranges from 30.5% in Nigeria, 43.4% in Zimbabwe, 45.3% in Kenya, 45.5% in Mozambique, 53.9% in Zambia and 57.6 in Cameron. The two countries (Zambia and Mozambique); the experience of violence is significantly higher among women from non-poor (rich) than (poor and middle). Other two countries (Zimbabwe and Kenya); women from poor households are more likely to have ever experienced IPV than those from non-poor households.	Experience of violence cuts across all household poverty-wealth statuses and therefore may not provide enough explanation on whether household poverty necessarily serve to facilitate the ending of violence. These results suggest that eliminating VAW in SSA requires a comprehensive approach rather than addressing household poverty-wealth alone.
Abeya et al., 2012. Ethiopia [75]	To explore the community attitude, strategies women’s suggested measures to stop VAW	Cross-sectional	Women and men (FGDs)	Purposefully	115	NR	Most discussants perceived, IPV is accepted in the community in circumstance of practicing extra marital sex and suspected infidelity. The suggested measures for stopping or reducing women’s violence focused on provision of education for raising awareness at all level using a variety of approaches targeting different stakeholders.	More efforts are needed to dispel myths, misconceptions, traditional norms and beliefs of the community. There is a need of amending and enforcing the existing laws and formulating the news policy.
Bazargan-Hejazia et al., 2013. Malawi [76]	To examine the lifetime prevalence of different types of IPV and its association with age, education, and residence	Cross-sectional	Women (15–49 years)	two-stage systematic sampling	8291	NR	The prevalence of emotional, physical and sexual IPV were 13, 20 and 13%, respectively. Women (15–19 years) were significantly less likely emotional IPV, women (25–29) were significantly more likely to report being physically abused (OR 1.35; CI: 1.05–1.73), and women (30–34) were significantly more likely sexual IPV, compared to women (45–49) (OR 1.40; CI: 1.03–1.90). Women who had no ability to read were less likely to report sexual IPV than their counterparts who could read a full sentence (OR 0.76; CI: 0.66–0.87).	The prevalence of different types of IPV in Malawi appears slightly lower than that reported for other countries in SSA. Further studies are needed to assess the attitudes and behaviors of Malawi women towards acceptability and justification of IPV as well as their willingness to disclose it.
Zacarias et al.2012 Mozambique [77]	To examine the occurrence, severity, chronicity and predictors of IPV	Cross-sectional	Women (15–49 years)	Consecutive case	1442	96.1%	The overall IPV during the past 12 months was 70.2%. Physical, psychological and sexual violence were the common IPV in Mozambique. Almost one fourth of women experienced combination of the three type of IPV.	Controlling behaviors over partner, co-occurring victimization and childhood abuse were more important factors.
Meekers et al., 2013, Bolvia [78]	To examine the relationship between IPV and mental health	Cross-sectional survey	Women (15–49 years)	Multistage sampling	10,119	NR	Life time physical and psychological IPV were 71.7 and 42.4%, respectively. Current IPV was 47%. Of these, physical, psychological and sexual IPV were 19.2, 21.1 and 6.9%, respectively.	It showed that mental health service is need for victims of IPV.
Abeya et al., 2011. Ethiopia [79]	To investigate the prevalence, patterns and associated factors of intimate partner violence against women in Western Ethiopia	Cross-sectional	Women (15–49 years)	Multistage systematic sampling	1540	96.3	Lifetime, current and concurrent IPV were 76.5, 72.5 and 56.9%, respectively. Rural residents (AOR 0.58, 95% CI 0.34–0.98), literates (AOR 0.65, 95% CI 0.48–0.88), female headed households (AOR 0.46, 95% CI 0.27–0.76); older women (AOR 3.36, 95% CI 1.27–8.89); abduction (AOR 3.71, 95% CI 1.01–13.63), polygamy (AOR 3.79, 95% CI 1.64–0.73), spousal alcoholic consumption (AOR 1.98, 95% CI 1.213.22), spousal hostility (AOR 3.96, 95% CI 2.52–6.20), and previous witnesses of parental violence (AOR 2.00, 95% CI 1.54–2.56) were factors associated with an increased likelihood of lifetime IPV.	Three out of four women experienced at least one incident of IPV in their lifetime. This needs an urgent attention at all levels of societal hierarchy including policymakers, stakeholders and professionals to alleviate the situation.
Koenig M. et al. 2003. Ugnada [6]	To examine individual risk factors associated with recent IPV and community attitudes	Cross-sectional survey	Women (15–49 years)	Cluster sampling	5109	NR	Overall, 40.1% of women had ever experienced psychological IPV and 30.4% of women had ever experienced physical threats or violence. The male partner’s alcohol consumption and his perceived human immunodeficiency virus (HIV) risk in increasing the risk of IPV.	Little progress in reducing levels of IPV is likely to be achieved without significant changes in prevailing individual and community attitudes toward IPV.
Wandera et al.2015 Uganda [80]	To investigate the association between IPSV and partner controlling behaviors	Cross-sectional survey (DHS 2011)	women (15–49 years)	Multistage cluster sampling	1307	NR	IPV was 27%. Women’s IPV experience was higher whose partner were jealous if they talked with other men, if accused them of unfaithfulness, if their partner did not permit them to meet with people, if their partner tried to limit contacts, got drunk, and women afraid of their partner.	Interventions addressing IPSV should be place more emphasis on reducing partners controlling behaviors and the prevention of problem drinking.
Deyessa N et al., 2009, Ethiopia [81]		Cross-sectional	Women (15–49 years)	SRS	1994	94.3%	The lifetime prevalence of any form of IPV was 72.0%. Physical violence was 49.5%.	Recommend public health strategies, interventions and service provision
Valladares E et al., 2005. Nicaragua [82]	To estimate the prevalence and characteristics of partner abuse during pregnancy	Cross-sectional	Women (15–49 years)	Cluster sampling	478	99.8%	The prevalence of emotional, physical, sexual and concurrent IPV were 32.4, 13.4, 6.7 and 17%, respectively. Factors such as women’s age below 20 years, poor access to social resources and high levels of emotional distress were independently associated with violence during pregnancy.	Although these women have poor access to social resources and high levels of emotional distress, they are rarely assisted by the health services.

*RR* Response Rate, *NR* Not Reported

**Table 2:** description of the interventional studies

Author, country	Study aim	Design	Population	Sampling procedure	sample	Intervention	Main findings	Key conclusion and recommendation
Abramsky et al.2014. Uganda [83]	To assess the community-level impacts of SASA! a community mobilization intervention to prevent violence and reduce HIV-risk behaviors	Cluster RCT	Women (15–49 years)	SRS	Baseline = 1583 Post-line = 2532	Control: existing service Intervention: SASA: Community mobilization(start, awareness, support and action)	The intervention was associated with significant lower social acceptance of IPV among women (ARR, 0.54, 95% CI: 0.38–0.79) and lower acceptance among men (0.13, 95% CI: 0.01, 1.15); significantly greater acceptance that a women can refuse sex among women (1.28, 95% CI:1.07, 1.52) and men (1.31,95% CI:1.00 to 1.7); 52% lower physical IPV (0.48, 95% CI:0.16,1.39); and lower levels of sexual IPV (0.76,95% CI: 0.33 to 1.72). IPV was more likely to receive supportive community responses. Sexual concurrency was significantly lower (0.57, 95% CI: 0.36, 0.91).	Community mobilization program on the social acceptability of IPV, past year prevalence of IPV and level of sexual concurrency archived important community impact and now delivered n control communities and replicate in other countries.
Gupta et al.2013. Cote d’IVoire [84]	To evaluate the incremental impact of adding gender dialogue groups to an economic empowerment group savings program on level of IPV	RCT	Women (15–49 years) and men	Simple random sampling	934	Control: VSIA Intervention: combined (VSLA+GDG)	Slightly lower odds of reporting past year physical and or sexual IPV in the combined group than VSAL alone (OR, 0.92, 95% CI: 0.58, 1.47). Women in the combined group were significantly less likely to report economic abuse than control (OR, 0.39, 95% CI: 0.25, 0.60). Acceptance of wife-beating was significantly reduced on intervention group (OR: −0.97, 95% CI: −1.67, − 0.28).	Combined intervention significantly reduce economic abuse and justified wife beating. But, no significant reductions on physical and or sexual IPV or sexual IPV alone.
Pulerwitz J et al., 2015. Ethiopia [85]	assessed the effects of a community-based project in Ethiopia that worked with young men to promote gender-equitable norms and reductions in IPV	Quasi-experimental	young people (15–24 years)	Randomly assigned	809	Control: existing service Active comparator: community education Intervention: combined (group education and community education)	Participants in the GE + CE intervention were twice as likely (*P* < .01) as those in the comparison group to show increased support for gender-equitable norms. Also, the percentage of GE+ CE participants who reported IPV toward their partner decreased from 53 to 38% between baseline and end line, and the percentage in the CE-only group decreased from 60 to 37%; changes were negligible in the control group.	Promoting gender equity is an important strategy to reduce IPV
Falb LK, et al., 2015. Cote d’IVoire [86]	To assess treatment heterogeneity based on child marriage status for an intervention seeking to reduce IPV	RCT	Women (15–49 years)	Random sampling	682	Control: VSIA Intervention: combined (VSLA+GDG)	For child brides; there were no statistically or marginally significant decreases in physical and or sexual IPV. The odds of reporting economic abuse in the past year were lower in the intervention arm for child brides relative to control group child brides (OR, 0.33, 95% CI: 0.13, 0.85). For non-child brides; women were less likely to report physical and or sexual IPV (OR, 0.54, 95% CI: 0.28, 1.04), emotional violence (OR, 0.44; 95% CI: 0.25, 0.77), and economic abuse (OR, 0.36, 95% CI: 0.20, 0.66) in the combined intervention arm than savings only groups.	Intervention participants with a history of child marriage may have greater difficulty benefiting from interventions that seek to reduce IPV.
Krishnan S. et al., 2012. Tanzania [87]	Examined men’s and women’s attitudes about IPV, relationship power, and sexual decision making and couples’	RCT	Couples	Random sampling	567	Intervention: Conditional cash transfers (CCT) promoted safe sex	Women who reported that violence was ever justified if a woman refuses sex were more than twice as likely to report IPV (aOR = 2.29,95% CI:1.65–3.17). Furthermore, women were less likely to report IPV when both partners shared sexual decision making (aOR = 0.70, 95% CI: 0.5–0.98), as compared to women’s partner controlled sexual decision making. Notably, women were less likely to report IPV when both partners had equal power (aOR = 0.43, 95% CI: 0.21–0.89) or they controlled more power (aOR = 0.91, 95% CI: 0.28–2.94).	RESPECT study indicate that concerted efforts to reduce IPV and promote gender equity have the potential to make a positive difference in the relatively short term.
Wagman AJ. et al.,2015. Uganda [88]	assess whether provision of a combination of IPV prevention and HIV services would reduce IPV and HIV incidence	Cluster RCT	Women (15–49 years)	Random sampling	11,448 individuals	standard of care HIV services plus a community-mobilization intervention the Safe Homes and Respect for Everyone (SHARE) Pro	Compared with control groups, the SHARE intervention groups had fewer self-reports of past-year physical IPV (16%) in control groups vs. (12%) in intervention groups; aPRR 0·79, 95% CI 0·67–0·92) and sexual IPV (13%) to (10%); 0·80, 0·67–0·97). Incidence of emotional IPV did not differ (20% vs 18%); 0·91, 0·79–1·04). SHARE had no effect on male-reported IPV perpetration.	SHARE could reduce some forms of IPV towards women and overall HIV incidence, possibly via reduction in forced sex and increased disclosure of HIV results.
Green PE, et al., 2015. Uganda [89]	To assess the effect of successful poverty alleviation on women empowerment and intimate partner relationship	Cluster RCT	Women (15–49 years)	Random sampling	1800	5 days business advice, 150USD and supervision	The program doubled the business ownership and incomes. It showed small increases in marital control, self-reported autonomy and quality of intimate partner relationship), but essentially no change in IPV and no effects on women’s attitude towards gender-norms and a non-significant reduction in autonomy.	Increasing women’s earnings has no effect on IPV.
Abramsky et al., 2016 Uganda [90]	To explore the community mobilization intervention to prevent VAW achieved community-wide reductions in physical IPV	Cluster RCT	Women aged 18–49 years	Cluster sampling	baseline = 1583 Endline = 2532	Control: existing service Intervention: SASA: Community mobilization(start, awareness, support and action)	SASA was associated with reductions in women’s current physical IPV (0.48, 95% CI 0.16–1.39), as well as men’s perpetration of IPV (0.39, 95% CI 0.20–0.73). Community-level normative attitudes were the most important mediators of intervention impact on physical IPV risk, with norms around the acceptability of IPV explaining 70% of the intervention effect on women’s experience of IPV and 95% of the effect on men’s perpetration.	It highlights the important role of community-level norm-change in achieving community-wide reductions in IPV risk.

### Data synthesis

The pooled estimate of domestic VAW was computed using the Comprehensive Meta-Analysis (CMA) software [91]. Substantial heterogeneity was assumed to be I^2^ (> 75%) [92, 93]. Potential publication bias was checked through visual assessment of the funnel plot [94, 95]. The random effects model [96] was used to moderate the sample size variation which might have had an influence on the pooled estimate. In addition, sub-group meta-analysis was performed by study settings and types of domestic VAW (lifetime, current, psychological, physical and sexual violence). Furthermore, the associated factors with VAW were qualitatively synthesized according to the ecological framework model (individual, relationship, community and societal factors) [97].

## Results

### Intimate partner violence in LLMICs

#### Lifetime intimate partner violence

**Fig. 2:**
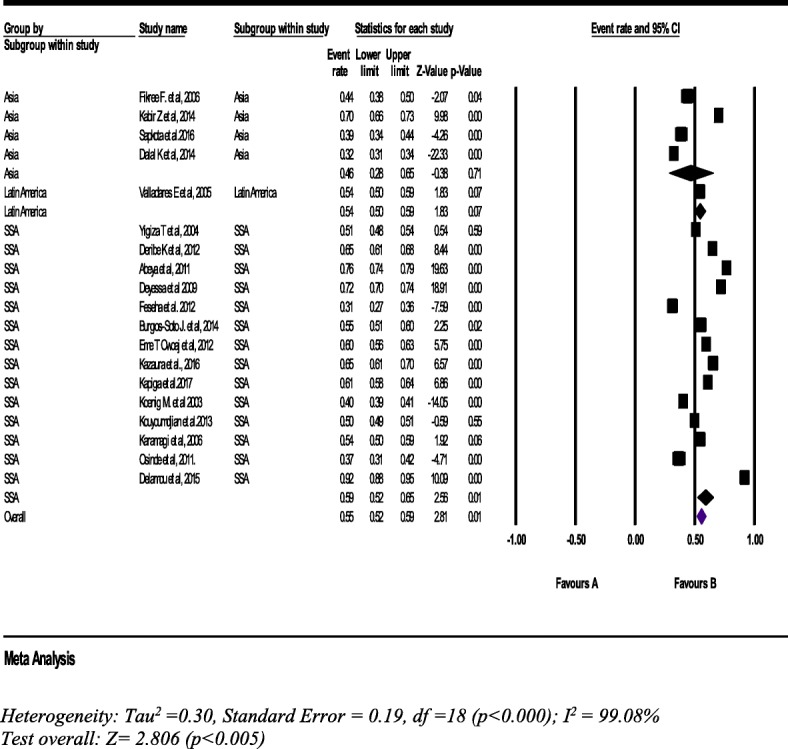
Forest plot of the lifetime intimate partner violence in LLMICs (*n* = 19)

Nineteen studies with a sample of 35,974 women (15–49 years), the pooled estimate of lifetime IPV was 55% (95% CI: 52, 59%). IPV in sub-Saharan Africa (SSA) (14 studies) and Asian countries (4 studies) were 59% (95% CI: 52, 65%) and 46% (95% CI: 28, 65%), respectively (Fig. 2).

#### Physical intimate partner violence

**Fig. 3:**
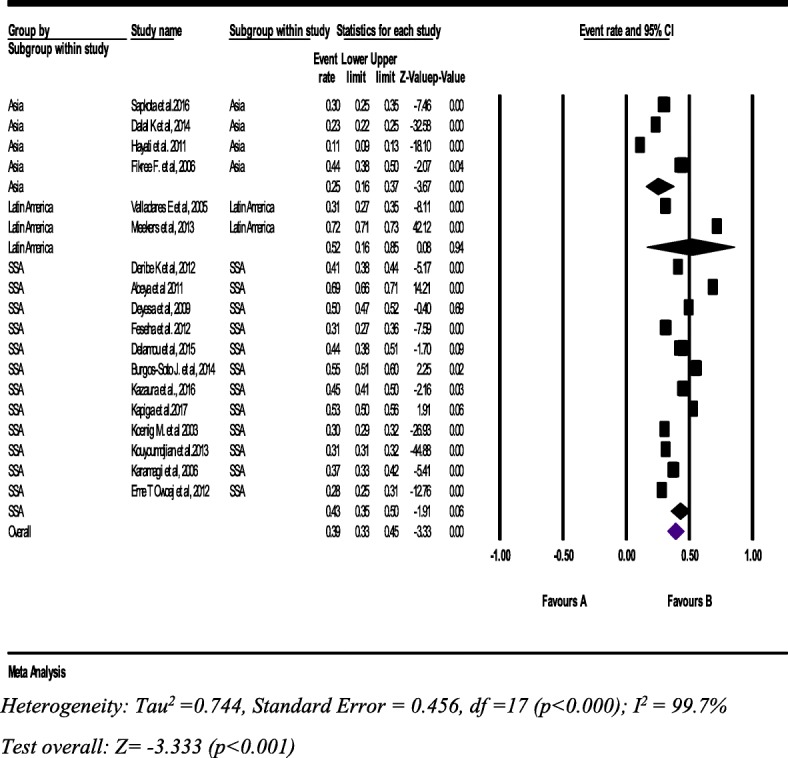
Forest plot of the Lifetime physical intimate partner violence against women (*n* = 18)

From 18 studies with a sample of 44,664 women (15–49 years, the pooled prevalence of lifetime physical IPV was 39% (95% CI: 33, 45%). Furthermore, lifetime IPV in SSA was 43% (95% CI: 35, 50%) (Fig. 3).

#### Psychological intimate partner violence

**Fig. 4:**
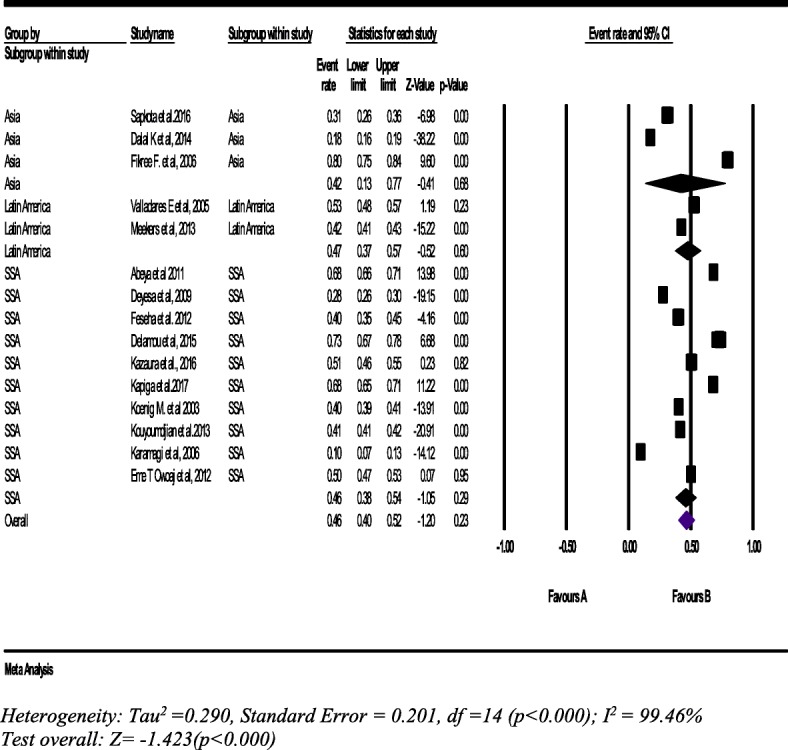
Forest plot of the lifetime psychological intimate partner violence against women (*n* = 15)

From 15 studies with a sample of 42,600 women (15–49 years), the pooled prevalence of lifetime psychological IPV was 46% (95% CI: 40, 52%). The sub-group analysis shows consistent findings with the overall pool prevalence across the regions (Fig. 4).

#### Sexual intimate partner violence

**Fig. 5:**
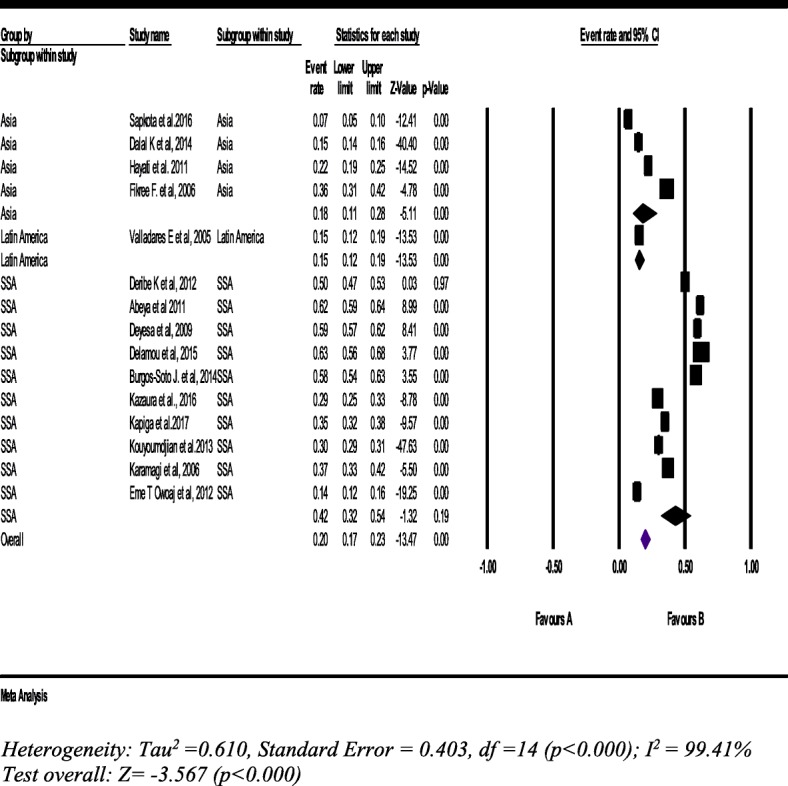
Forest plot of the lifetime sexual intimate partner violence against women (*n* = 15)

From 15 studies with a sample of 29,127 women (15–49 years), the pooled prevalence of sexual IPV was 20% (95% CI: 17, 23%). The lifetime sexual IPV was the highest in SSA [42% (95% CI: 32, 54%)] (Fig. 5).

#### Current intimate partner violence

**Fig. 6:**
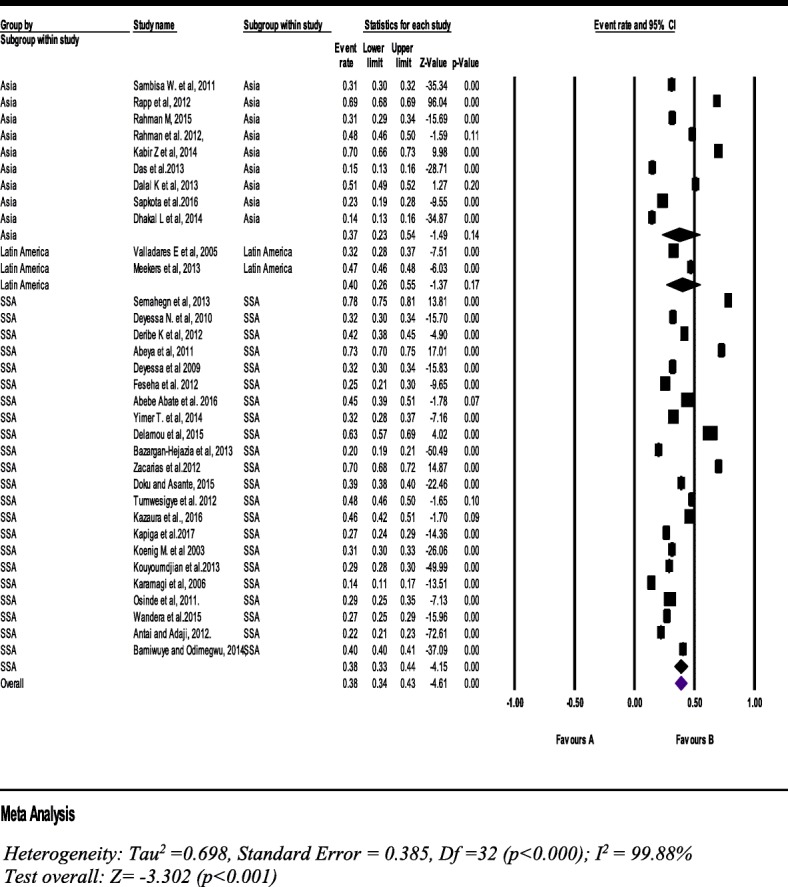
Forest plot of the current intimate partner violence against women (*n* = 33)

Thirty three studies with a sample of 216,043 women (15–49 years), the pooled prevalence of IPV was 38% (95% CI: 34, 43%). The prevalence in SSA is almost similar with the pooled prevalence (Fig. 6).

### Types of current intimate partner violence

#### Physical intimate partner violence

**Fig. 7:**
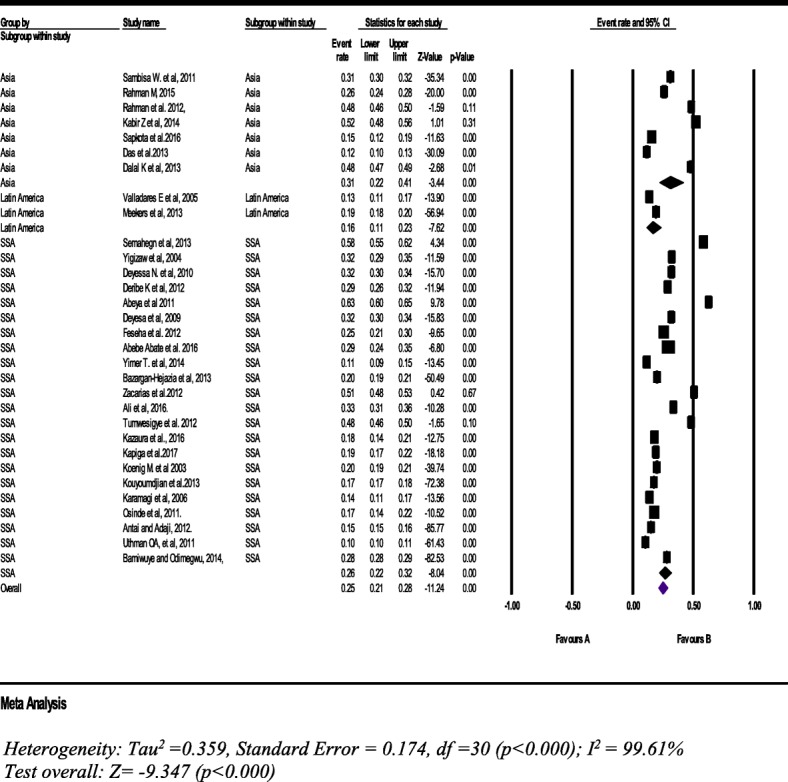
Forest plot of the current physical intimate partner violence against women (*n* = 31)

From thirty one studies with a sample of 141,820 women (15–49 years), the pooled prevalence of physical IPV during the past 12 months was 25% (95% CI: 21, 28%). The subgroup analysis of seven studies in Asian countries was 31% (95% CI: 22, 41%) (Fig. 7).

#### Psychological intimate partner violence

**Fig. 8:**
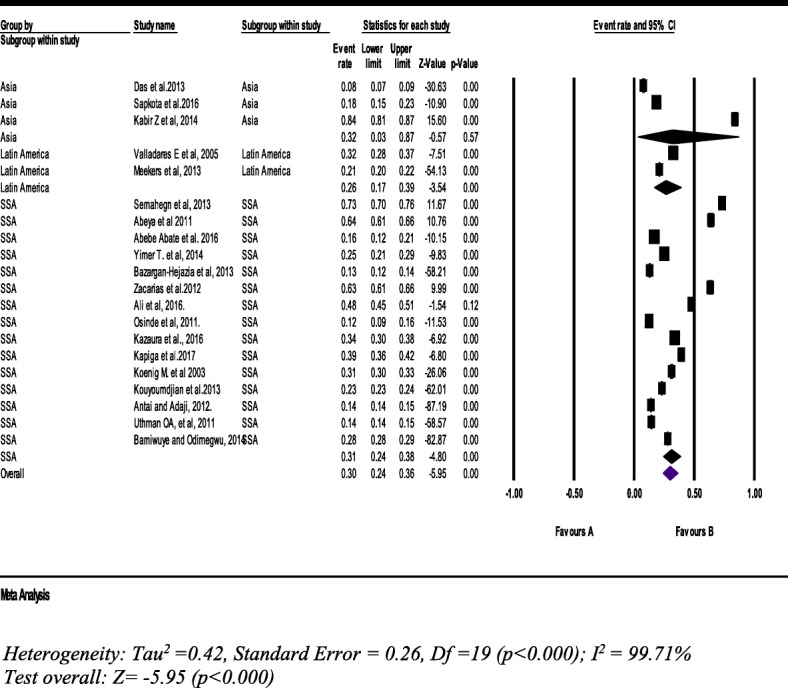
Forest plot of the current psychological intimate partner violence against women (*n* = 20)

From 20 studies with a sample of 115,798 women (15–49 years), the pooled prevalence of psychological IPV was 30% (95% CI: 24.0, 36%) (Fig. 8).

#### Sexual intimate partner violence

**Fig. 9:**
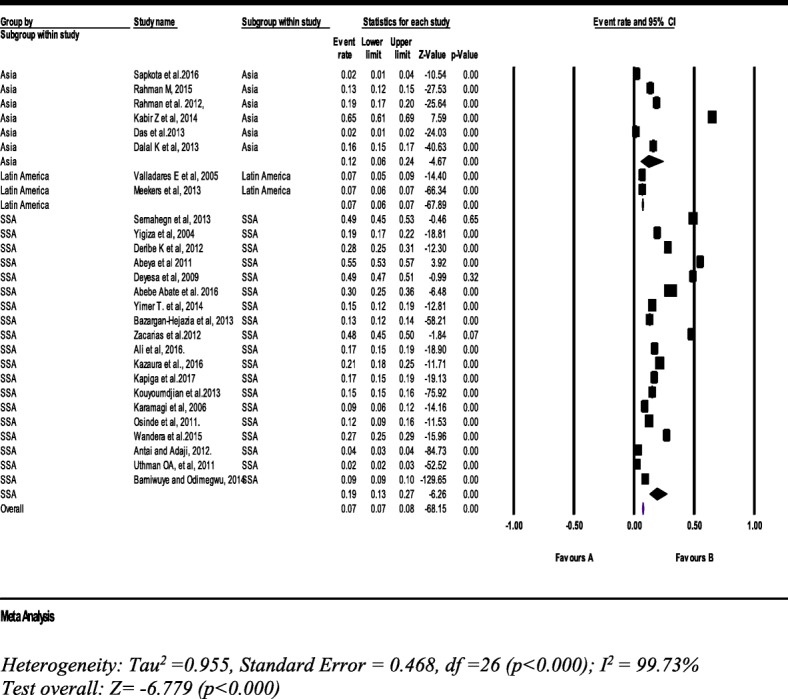
Forest plot of the current sexual intimate partner violence against women (*n* = 27)

From 27 studies with a sample of 124,739 women (15–49 years), the pooled prevalence of current sexual IPV was 7% (95% CI: 7, 8%). However, the subgroup pooled prevalence was a bit higher than the overall pooled prevalence. It was 19% (95% CI: 13, 27%) in SSA countries (Fig. 9).

#### Concurrent intimate partner violence

**Fig. 10:**
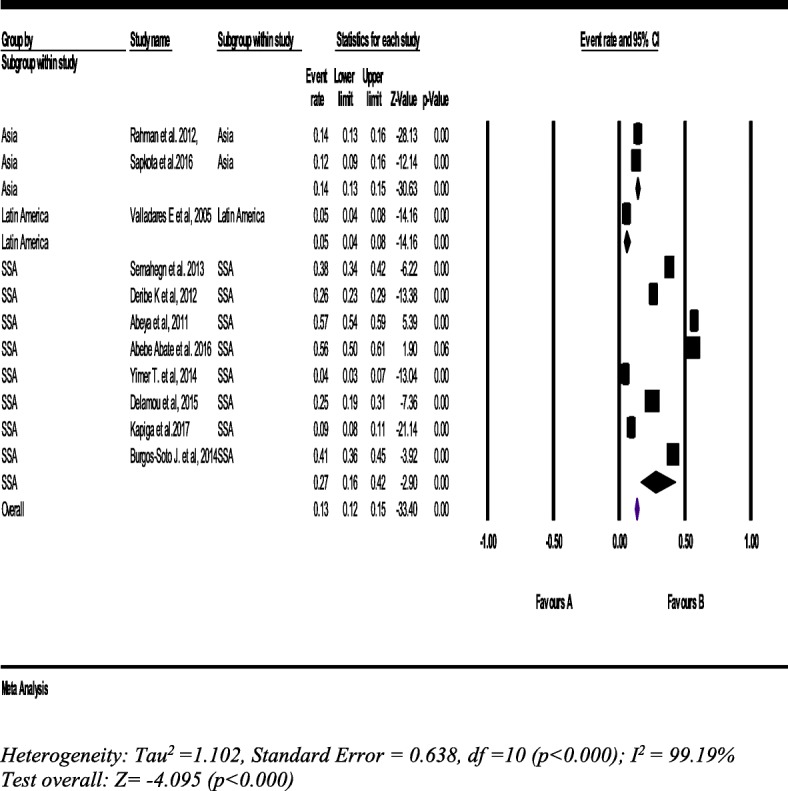
Forest plot of the pooled prevalence of concurrent intimate partner violence against women (*n* = 11)

From eleven studies with a sample of 8315 women (15–49 years), the pooled prevalence of women’s experience of IPV concurrently was 13% (95% CI: 12, 15%). In the meantime, the prevalence of concurrent IPV in SSA was 27% (95% CI: 16, 42%) which is two times higher than the overall pooled IPV prevalence in LLMICs (Fig. 10).

### Contributing factors of domestic violence against women

#### Individual level factors

##### Socio-demographic factors

The socio-demographic characteristics of the couples were identified as factors associated with IPV. In six studies, place of residence was one of the factor associated with IPV whereby women living in rural area were more likely to experience IPV than urban dwellers [25, 52, 61, 62, 65, 79]. Similarly, in two studies, women’s religion was a factor associated with IPV, such that women belonging to Islam were more likely to experience IPV than non-Muslim [65, 70]. In addition to Muslim women, women belonging to the traditional religion (worshipers) and being in other faith(s) were more likely to accept physical IPV than women belonging to Christianity [52].

##### Age

Differences between women’s and their husbands’ age had an inconsistent relationship with the experience of IPV. In three studies, physical IPV was significantly associated with women’s age and age at first marriage [40, 48, 56]. Women’s age 20 years and younger was a risk factor associated with IPV [82]. In one study, women who married at age 15 or young were four times more likely to experience IPV than women got married older than 15 years [64]. While women (15–19 years) were less likely to report emotional IPV, women (25–29 years) and women (30–34 years) were more likely to report being physical and sexual IPV, respectively than women (45–49 years) [76]. Nevertheless, in two studies, older age women (35–49 years) were three times more likely to report lifetime and current IPV than women (15-19 years) [53, 79]. In addition, the age of husbands was a positive predictor of IPV [40, 49], but sexual IPV was associated with husbands younger than 35 years [51].

##### Education

Women’s lifetime IPV remained significantly associated with women’s level of education. In six studies, uneducated or primary educated women had almost double the prevalence rate of any form of IPV who attended secondary or higher education [6, 48, 52, 53, 60, 65]. In five studies, the higher the women’s educational level, the lesser the likelihood of experiencing physical IPV. Women with higher education than their husbands were less likely to experience IPV than women with equal or less education than their husbands [40, 55, 56, 62, 71]. In two studies, women in equally high educated marriages revealed the lowest likelihood of experiencing IPV. Education was found to be significantly protective from IPV, for both women as victim and men as perpetrator [55, 79]. Furthermore, the educational status of husbands was associated with women’s experience of less IPV and less violent behavior to their wives [27, 50, 53, 54, 58]. On the other hand, women with no education were about three times more likely to approve (accept) IPV than women with secondary or higher education [52]. IPV remained higher and even increased in the case of women with secondary to vocational levels of education as compared to those with a higher level of education [57, 66].

##### Occupation

In two studies, women who were engaged in manual labor (farming) were three times more likely to be exposed to IPV than women with non-farming occupations [66, 70]. In one study, women who has been in the poorest microfinance group under supported by the microfinance programmes in Bangladesh have no significant change on IPV exposure, except some improvement on economic empowerment [65]. Likewise, the women whose partners are employed (earned in cash) were 93% less likely to experience physical IPV than those women whose partners were farmers (earned in kind) [70]. Likewise, in three studies, women were economically dependent, and or did not have their own means of earnings and controlling their earnings were more likely to report sexual IPV than their counterparts [49, 51, 72]. Similarly, women heading business or engaged in different types of jobs were 50% less likely to report lifetime IPV than women have not heading business (almost jobless women) [79]. On the contrary, housewives were less likely to be experienced sexual IPV than women who have a job different from housewives [68].

##### Childhood witness

Domestic IPV was significantly associated with women and their husband childhood wittiness of parental violence. Although the strength of association varies (ranged from four to thirteen times), those women whose mothers were hit by their fathers during their childhood were more likely to report lifetime and current IPV than their counterparts [25, 53, 61, 72, 79]. Likewise, in some studies, women whose husbands were beaten by someone in their family during their childhood were two times more likely to report experiences of IPV than those who were not beaten during childhood [53, 72, 79]. In addition, women’s exposure to physical IPV was 5 to 6 times more likely to be higher on women whose husband having witnessed that their mothers being beaten by their fathers than women whose husband had no history (witness) of maternal IPV [51, 66, 70]. Furthermore, in one study, IPV was associated with women who had a history of sexual abuse during childhood, adolescence or even an early age of first sex [60].

##### Husbands’ controlling behaviors and mental health condition

IPV was higher among women who were afraid of their husbands [53, 80]. Women whose husbands had controlling, hostile and or rude behaviors were almost three to four times more likely exposed to any type of IPV than their counterparts [47, 53, 68, 77, 79]. Women who had high levels of emotional distress were associated with IPV [82]. Furthermore, women whose husbands previously engaged in physical fight were 3.5 times more likely to experience IPV than others. In a similar way, women with an unhealthy mental status were two times more likely to experience IPV than women with a healthy mental status [47].

##### Husband alcohol consumption

Women whose husbands’ drank alcohol were more likely to experience IPV than women whose husbands did not drink alcohol. As we found from several studies, husbands’ alcohol consumption is the most commonly reported factor associated with IPV [6, 25, 49–51, 56, 60, 62–64, 66, 70, 79, 80]. Furthermore, in one study, IPV was significantly associated with husband’s use of psychoactive substances [66].

##### Pregnancy status

In three studies, pregnant women with high parities had a higher probability of experiencing lifetime IPV than non-pregnant women [48, 49, 66]. Furthermore, in one study, women whose pregnancies were undesired by their partners was six times more likely to have risk of current IPV than pregnancy desired by partner [64]. Likewise, in two studies, the likelihood of women’s experience of lifetime IPV was consistently higher as the number of children increased [48, 54].

#### Relationship factors

##### Women’s decision making status

Decision making power was a predictor of IPV [49]. Generally, the odds of IPV was less by 50% for women who had an equal say in household decision-making. Sexual violence was 35% less likely to occur among women who had a share in household decision-making [25]. Interestingly, the probability of women being physically abused decreased by 8.2% for those women whose husbands dominated household decision-making, whereas wife-dominance in household decision-making had a marginal effect on physical violence [54]. However, women who decided on spending their own or husband’s earning with a joint decision-making approach in view of their own healthcare utilization, large household purchase, or contraceptive use were less likely to be victims of IPV than women who made decisions by themselves [53].

##### Infidelity and lack of satisfaction in marriage

In four studies, women whose husbands had engaged in extramarital sex or had multiple sexual partners (unfaithful) were two times more likely to be at risk of IPV than their counterparts [51, 62, 72, 80]. Yet, exposure to physical IPV was associated with being unfaithful. Women who agreed that a woman was obliged to have sex with her husband had a lower risk of exposure to physical IPV than those who disagreed [51]. Women who had poor relationships with their husbands were 2.6 times more likely to experience physical IPV [58]. Furthermore, women who had a satisfaction in their marriage were noted to face a low risk of IPV [62]. Sexual IPV was higher among women whose partner were jealous if they talked with other men, suspected them of unfaithfulness, did not permit them to meet even female friends, limit their contact with family [80]. Those women who did not believe a wife could do anything if a husband wanted a girlfriend were three times more likely to be exposed to physical IPV [68]. In the same view, women who could refuse sex with their partners or ask their husbands to use condoms were two times more likely to be victims of IPV than their peers who could not [53]. Women who had worries on issues about their daily activities and did not discuss them with their partners were more likely to experience IPV than women who did discuss their issues [66].

##### Type of marriage

In five studies, the type of marriage was significantly associated with IPV. Women who were married or cohabitated by abduction; women married to distant relatives; women with a polygamous partner or in a polygamous marriage; payment of dowry and marriage undergone without ceremony were more likely to experience IPV than their counterparts [27, 48, 50, 61, 79].

##### Duration of marriage

In two studies**,** this factor was significantly associated with higher experience of physical abuse. Women who were married for 5–9 years (OR, 3.8) or ten or more years (OR, 3.7) were at higher risk of being abused than women who were married for less than 5 years [48, 60].

##### Wealth index and economic status

IPV and wealth index had an association but an inconsistent relationship across the wealth quintiles. In six studies, women belonging to the poorest wealth index categories were most likely to be exposed to IPV than women in the rich wealth index [51, 52, 54, 63, 65, 74]. Other studies, microfinance program membership was associated with a two-three-fold increase in exposure to IPV [65], as were family and financial problems associated with IPV [66, 79].

#### Community level factors

##### Presence of the traditional gender norms

In two studies, women’s exposure to IPV were more likely to be high in communities who adhered to traditional patriarchal gender norms or beliefs and supported (accepted) attitudes towards wife beating [51, 54]. Meanwhile, women who agreed that “a good wife obeys her husband” and/ or “a man should show who the boss is” were more likely to experience sexual IPV than women who disagreed. In addition, women who agreed that a woman had no reason to refuse sex with her husband were three times more likely to be exposed to IPV as compared to who agreed for some reasons [51]. In addition, women who worked outside the home but whose husbands did not make enough money had an increased risk of IPV by 5.2% than women whose husbands made enough money [54].

##### Community attitude towards wife-beating

Communities whose attitudes supported IPV [66] by thinking that justified wife-beating is acceptable [60], or encouraged societal gender beliefs or norms [54], reflected relationship control, and relationship inequalities [40, 51] were significantly associated with women’s experience of IPV.

### What were the key recommendations from the studies?

#### Transformation of community’s traditional gender norms

In seven studies, undertaking a massive and intensive information, education and communication (IEC) approach is a recommended strategy on transforming a community’s culture and traditional gender norms in order to enhance gender equality [25, 27, 38, 47, 49, 51, 61]. Similarly, in eight studies, there is a need to employs comprehensive and culturally acceptable approaches including medical (psychiatric) counseling, community mobilization, gender advocacy and effective development of IEC to dispel myths, misconceptions, negative traditional norms and beliefs, gender inequality and to reduce the costs of IPV [6, 40, 54, 68, 70, 72, 75, 79].

#### Human right based approach

In one study, community level awareness of human rights as well as advocacy for women’s rights is crucial [67]. In four studies, more investments in IPV prevention strategies are needed to address the intergenerational transfer of deeply entrenched cultural-norms which support male dominance and gender inequality [57, 66, 73, 90].

#### Stakeholder engagement

The urgent attention of policymakers, stakeholders, professionals and other concerned bodies is needed at all levels of society. Stakeholders should design interventions targeting behavioral and social factors which can help to prevent IPV [79]. Likewise, resources should be mobilized by policy-makers, public health experts, researchers and other stakeholders to prevent IPV [48, 59, 63, 72]. Advocacy is very important and can be done as religious institutions, media, government and non-governmental associations encourage gender equality by [66, 67, 70, 73].

#### Policy formulation and legal framework

The issue of gender equality, women’s rights and legal sanctions need due attention during policy formulation and endorsement of laws to prevent VAW are crucial. In many instance of VAW, the punishment to perpetrators were light and not commensurate with the offence victims filed. The law should be more responsive to VAW to help address this challenge [25, 68]. Perhaps, survivors are encouraged to disclose their experience of IPV to people who are in position or have an autonomy, and implementing the existing law for punishment [70]. Policymakers should take immediate action to break hierarchical barriers between spouses, and promote gender equality while amending the existing laws or formulating new policies [52, 53, 75]. However, evidence based efforts are needed to re-enforce legal rights or existing laws and policies and ensure their effective implementation to prevent and respond to VAW [54, 57, 67].

#### Women empowerment

Six studies have recommended that building women’s capacity through education, employment, income and other economic opportunities, and addressing imbalance of power between men and women are crucial to prevent IPV [25, 47, 52, 53, 56, 79]. Likewise, promising public health strategies (increasing awareness of the consequences of IPV, strengthening the self-esteem of women and girls and promoting equity in marital relationships) are needed to change attitudes towards gender inequality, and are essential to avert IPV [47, 70, 79]. Furthermore, addressing household poverty-wealth comprehensively [74], enhancing the safety of women, promoting fertility control methods and women’s reproductive health service [40] are some other recommended interventions to prevent and control IPV.

#### Intervention integration

Innovative strategies are needed to provide support and counselling to IPV survivors, who are rarely assisted by health care professionals [82]. Research findings have strongly recommended that IPV prevention interventions be integrated with community health programs; reproductive health and other health services to be more comprehensive, close to household level and accessible to IPV survivors [27, 49, 53, 67, 70]. Integration may help to enhance medical screening of survivors for STI including HIV, provide male partner counseling and other health care support. Furthermore, the authors [38, 48, 67, 78] recommended that reproductive health service providers should be encouraged to advocate for IPV and mental health screening during antenatal care.

#### Engaging men and other influential persons on IPV prevention

Research findings have proposed that community programs that have a couple-centered approach are needed to promote non-violent masculinity values and norms [47, 51, 58]. Moreover, working with men is a win-win-approach to prevent IPV. Efforts are needed to focus societal, community, relationship and individual level approaches which engage men to promote men’s non-violent behavior and gender equality and to minimize infidelity [51, 62, 67]. In addition, interventions addressing IPV should place more emphasis on reducing partners’ controlling behaviors and to prevent men’s alcohol drinking habits [56, 62, 66, 80]. Furthermore, involving mother-in laws would be a significant move [47]. Also, community health workers could be active players in raising community awareness about IPV [27, 49].

### What are the proven evidence?

We systematically selected eight interventional studies that had been conducted in LLMICs [83–90]. The detail of GRADE for the summary of evidence for different outcomes is attached as an Additional file 6. In two studies, the social accepting attitude of IPV was lowered by community mobilization intervention. Women’s attitude towards sex refusal when necessary was 1.3 times more likely higher in the community mobilization intervention than control group. Likewise, current physical, sexual and concurrent IPV were lowered in the intervention group by 52, 24%, and 435 respectively than the control group. In addition, women experiencing IPV in intervention communities were more likely to receive supportive community responses. [83, 90]. In one study, the community-level normative attitude towards physical IPV and IPV acceptability norms were improved in the intervention group than the control. In addition, men’s suspicion of their partner for infidelity and communication about sex were improved [90]. Similarly, in one study, community engagement and group education combined interventions reduced IPV almost by 20% while the CE-only group reduced it by 23%, and enhanced gender equitable norms [85].

In the two interventional studies which is mainly focused on women’s economic empowerment through a village loan and saving association approach, women in the combined groups were significantly less likely to report economic abuse than control group (OR, 0.39, 95% CI: 0.25, 0.60) [84, 86]. In one study, while attitude towards refusal of sex did not significantly change, women in the combined (VLSA and GDG) intervention group reported a lower experience of current IPV and also acceptance of justified wife-beating than VLSA alone, but it was not statistically significant [84]. In one study, women who got married when they were children were 46% less likely to report physical and or sexual violence in the combined intervention group than the control. The reduction was however, not statistically significant in the overall IPV [86].

Women’s attitudes about IPV and power relationships were associated with their IPV experience. In one study, women who report violence was ever justified if a woman refuses sex were two times more likely to experience IPV than control group. Furthermore, women in joint (both partners) sexual decision making relationships were 30% less likely to report IPV as compared to women whose partners controlled sexual decision-making. Notably, women were 57% less likely to report IPV when both partners had equal power [87]. However, women’s economic empowerment was doubled for those women in the safe home and respect for everyone (SHARE) intervention group. Likewise, women in the intervention group were 20% less likely to experience IPV than those in the control group. Nevertheless, SHARE had no significant effect on emotional IPV, men’s behavior (perpetration) [88], overall IPV, women’s autonomy and women’s attitude towards gender norms [89].

## Discussion

This systematic review and meta-analysis determined the prevalence of domestic IPV and its types, associated factors, effective interventions and key recommendations to prevent domestic VAW. We found out that the pooled prevalence of lifetime IPV was 55% (95% CI: 52, 59%). Of these, lifetime physical, psychological and sexual violence were 39, 45 and 20%, respectively. Furthermore, the pooled prevalence of current IPV was 38% (95% CI: 33.0, 43%). Of these, prevalence of current physical, psychological and sexual violence were 25, 30 and 7%, respectively. In addition, the pooled prevalence of women’s experience of concurrent IPV was 13% (95% CI: 12, 15%). This finding is consistent with the WHO’s global estimates and multicounty study whose findings indicated that one-in- three women experienced domestic VAW in their lifetime [1, 98, 99].

As evidence shows that IPV was significantly associated with educational level, place of residence, economic status, having witnessed abuse during childhood, husbands’ having controlling behavior, husbands’ alcohol consumption, pregnancy status and parity. Notwithstanding, sexual violence was common among women who had husbands younger than 35 years. Furthermore, women in unfaithful relationships and unsatisfied marriages as well as women who married early and have experienced forced first sex, short duration of marital life, and poor mental health had a higher risk of experiencing IPV. In addition, the presence of traditional gender-norms and wife-beating accepting attitudes were linked with a high risk of domestic violence. This finding is also consistent with the ecological model formulated in 1998 [97, 100] which is very applicable in low and lower-middle income settings and is targeted at the deep rooted causes.

This systematic review found out that IPV prevention should focus on community culture or traditional gender-norm transformation; stakeholders’ engagement; women empowerment (capacity building); engaging men and other influential people (mother and/ or father in-laws, sister-in-law, neighbors); intervention should focus on service integration with other relevant sectors (mainly health sector for screening and other care and support); policy formulation and provision of legal framework and implementation of human right based approaches. This finding is consistent with other reviews that recommended focus on the structural drivers of unequal power in relationships to prevent VAW [98, 100–102].

We included observational and interventional studies. However, we interpreted the findings to meet our review objectives. Nevertheless, we did not mix-up the findings of the observational and interventional studies. Though, this systematic review and meta-analysis used relatively comprehensive search of the major databases; included both quantitative and qualitative studies; published and unpublished studies and sub-group analysis by setting and violence types, it had some limitations. One of the main limitations was the inconsistent definition of IPV across some studies. Hence, we used both IPV and domestic VAW synonymously. The second limitation of this systematic review and meta-analysis is that it did not carry out quantitative synthesis on the factors associated with domestic VAW due to the high heterogeneity between included studies. However, it is difficult to find absolutely homogeneous studies in terms of setting, method, analysis and interpretation.

### Implication of the review

Domestic violence against women is a common women’s life experience and mostly perceived as minor and socially tricky by many governments. However, its consequences have a devastating impact on national gross domestic product and costs much higher than the budget allotted for primary education. Hence, generating evidence on the prevalence, associated factors and identifying effective interventions applicable in poorly resourced settings is very crucial. This systematic review and meta-analysis can give critical insight about VAW, associated factors, and effective interventions. Therefore, concerned stakeholders can use the findings of this study as main evidence to inform policymakers, program designers and local planners construct and for implement policies to prevent IPV in LLMICs.

## Conclusion

Both lifetime and current domestic IPV are still high in LLMICs. More than half of the women in the studies had experienced lifetime domestic IPV, and almost one-third of the women had experienced current IPV. In addition, almost one-in-ten women experienced more than one type of IPV concurrently. Domestic IPV is a complex public health and human rights violation which is associated with factors at the individual, relationship, community and entire system level. However, most of the associated factors are preventable. Interventions integrating legal framework and programs that focus on transformation of traditional gender-norms are most important to prevent IPV. Community mobilization and awareness creation to transform gender-norm reduced IPV by half. However, the economic empowerment intervention had reduced some influences on women (reduced economic abuse), but it was not statistically significant in the prevention of IPV. Therefore, we suggest that researchers, program planners, policy makers, clinicians and other concerned stakeholders should invest in the implementation of gender-norms focused on community based interventions to prevent IPV.

## Additional files

Additional file 1: PRISMA checklist. (DOC 85 kb)

Additional file 2: **2-1** & **2-1-1**: Searching strategy on PubMed database. **2-2** Searching strategy on Medline database. **2-3** Searching strategy on EMBASE database. **2-4** Searching strategy on CNHAL database. (ZIP 2206 kb)

Additional file 3: Studies quality assessment using JBI critical appraisal checklist. (DOCX 24 kb)

Additional file 4: **4-1** Heterogeneity between studies illustration using funnel plot. **4-2** Risk of bias (ROB) assessment. (ZIP 69 kb)

Additional file 5: The extracted data stored on Microsoft Excel sheet for met-analysis. (XLSX 19 kb)

Additional file 6: GRADE for the summary of evidence for different outcomes. (DOCX 18 kb)

Additional file 7: Confirmation letter for funding. (PDF 1930 kb)

## Abbreviations

AOR: Adjusted Odds Ratio
CE: Community engagement
CI: Confidence interval
GDG: Gender Dialogue Group
IEC: Information, education and communication
IPV: Intimate partner violence
JBI: Joana Briggs Institute
LLMICs: Low and lower-middle income countries
SHARE: Safe Home and Respect for Everyone
SSA: Sub-Saharan Africa
TDR: Tropical Disease Research
VAW: Violence against women
VSLA: Village Save and Loan Association
WHO: World Health Organization
