# Supplementary material for: Are interventions focused on gender-norms effective in preventing domestic violence against women in low and lower-middle income countries? A systematic review and meta-analysis
Source: Reprod Health. 2019 Jul 1;16:93. doi: 10.1186/s12978-019-0726-5 (PMC6604322; doi:10.1186/s12978-019-0726-5)
Supplement: Supplementary file 2 — 2-1 & 2-1-1: Searching strategy on PubMed database. 2-2 Searching strategy on Medline database. 2-3 Searching strategy on EMBASE database. 2-4 Searching strategy on CNHAL database. (ZIP 2206 kb) [file 12978_2019_726_MOESM2_ESM.zip › additional file 2_ 4R1.pdf]

EBSCOhost

Library Logo

Search History/Alerts

Print Search History | Retrieve Searches | Retrieve Alerts | Save Searches / Alerts

Select / deselect all | Search with AND | Search with OR | Delete Searches | Re

	Search ID#	Search Terms	Search Options	Actions	
☐	S10	S7 AND S8	**Limiters** - Full Text<br>**Search modes** - Boolean/Phrase	**View Results** (101)	**View Detai**
☐	S9	S7 AND S8	**Search modes** - Boolean/Phrase	**View Results** (108)	**View Detai**
☐	S8	developing countries OR low and middle income countries OR least developed countries OR sub-Saharan countries OR African the south of Saharan countries OR under developed countries	**Search modes** - Boolean/Phrase	**View Results** (26,029)	**View De**
☐	S7	domestic violence agaisnt women OR intimate partner violence	**Search modes** - Boolean/Phrase	**View Results** (9,044)	**View Det**
☐	S6	(domestic violence against women OR intimate partner violence against women OR physical violence against women OR sexual violence against women OR psychological violence against women OR emotional violence against women OR verbal violence against women) AND (developing countries OR low and middle income countries OR poor resource setting OR least developed countries OR sub-Saharan countries OR African the south of Saharan countries OR limited resource settings OR under developed countries)	**Search modes** - Boolean/Phrase	**View Results** (14)	**View Details**
☐	S5	(Domestic violence against women OR	**Search modes** - Boolean/Phrase	**View Results** (12)	**View Details**

		intimate partner violence against women) AND (developing countries OR low and middle income countries OR poor resource setting OR least developed countries OR sub-Saharan countries OR African the south of Saharan countries OR limited resource settings OR under developed countries)		
☐	S4	(Domestic violence against women OR intimate partner violence against women) AND (associated factors OR contributing factors OR risk factors OR determinants OR predictors OR correlates OR influencing factors) AND (developing countries OR low and middle income countries OR poor resource setting OR least developed countries OR sub-Saharan countries OR African the south of Saharan countries OR limited resource settings OR under developed countries)	**Search modes** - Boolean/Phrase	**View Results** (7) | **View Details**
☐	S3	(Domestic violence against women OR intimate partner violence against women) AND (women aged 15 to 49 years OR women in the reproductive age OR childbearing aged women) AND (developing countries OR low and middle income countries OR poor resource setting OR least developed countries OR sub-Saharan countries OR African the south of Saharan countries OR limited resource settings OR under developed countries)	**Search modes** - Boolean/Phrase	**View Results** (1) | **View Details**
☐	S2	(domestic violence agaisnt women OR	**Search modes** - Boolean/Phrase	**View Results** (49) | **View Detail**

		intimate partner violence OR physical violence against women OR sexual violence against women OR psychological violence against women OR emotional violence against women OR verbal violence against women) AND (associated factors OR contributing factors OR risk factors OR determinants OR predictors OR correlates OR influencing factors) AND (developing countries OR low and middle income countries OR poor resource setting OR least developed countries OR sub-S ...		
☐	S1	domestic violence against women	**Search modes** - Boolean/Phrase	**View Results** (190) | **View Detai**

---

1. How Nurses in Johannesburg Address **Intimate Partner Violence** in Female Patients: Understanding IPV Responses in **Low-** and **Middle-Income Country** Health Systems.

   (includes abstract) Sprague, Courtenay; Hatcher, Abigail M.; Woollett, Nataly; Black, Vivian; Journal of Interpersonal **Violence**, Jun2017; 32(11): 1591-1619. 29p. (Article) ISSN: 0886-2605

   One in three women, globally, experiences **intimate partner violence** (IPV). Although 80% of the world's population resides in the **low**- and **middle-income countries** (LMICs), health system responses ...

   Subjects: Women's Health; **Intimate Partner Violence** Nursing; Victims Psychosocial Factors; Adult: 19-44 years; **Middle** Aged: 45-64 years; Female

   Cited References: (62)

   Linked Full Text PlumX Metrics

2. Exploring opportunities for coordinated responses to **intimate partner violence** and child maltreatment in **low** and **middle income countries**: a scoping review.

   (includes abstract) Bacchus, Loraine J.; Colombini, Manuela; Urbina, Manuel Contreras; Howarth, Emma; Gardner, Frances; Annan, Jeannie; Ashburn, Kim; Madrid, Bernadette; Levtov, Ruti; Watts, Charlotte; Psychology, Health & Medicine, 2017 Supplement; 22 135-165. 31p. (Article) ISSN: 1354-8506

   Intimate partner violence (IPV) and child maltreatment (CM) by a parent or caregiver are prevalent and overlapping issues with damaging consequences for those affected. This scoping review aimed ...

   Subjects: Intimate Partner Violence; Child Abuse

   Linked Full Text PlumX Metrics

3. [Systematic review of structural interventions for **intimate partner violence** in **low**- and **middle-income countries**: organizing evidence for prevention.]

(includes abstract) Bourey, Christine; Williams, Whitney; Bernstein, Erin Elizabeth; Stephenson, Rob; BMC Public Health, 11/23/2015; 15(1): 1-18. 18p. (journal article) ISSN: 1471-2458 PMID: 26597715 PMCID: PMC4657265

Background: Despite growing attention to **intimate partner violence** (IPV) globally, systematic evaluation of evidence for IPV prevention remains limited. This particularly is true in relation to **low**...

Subjects: Intimate Partner Violence Prevention and Control; Rape Prevention and Control; **Developing Countries**; Poverty Prevention and Control; Women's Rights; Sexism Prevention and Control; Male; Female

Show all 9 images

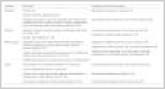
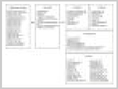
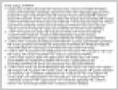
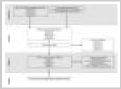

PDF Full Text PlumX Metrics

4. Barriers and Facilitators to Integrating Health Service Responses to **Intimate Partner Violence** in **Low**- and **Middle-Income Countries**: A Comparative Health Systems and Service Analysis.

(includes abstract) Colombini, Manuela; Dockerty, Colleen; Mayhew, Susannah H.; Studies in Family Planning, Jun2017; 48(2): 179-200. 22p. (journal article) ISSN: 0039-3665 PMID: 28422291

This systematic review synthesizes 11 studies of health-sector responses to **intimate partner violence** (IPV) in **low**- and **middle-income countries**. The services that were most comprehensive and inte...

Linked Full Text PlumX Metrics

5. Healthcare system responses to **intimate partner violence** in **low** and **middle-income countries**: evidence is growing and the challenges become clearer.

(includes abstract) Taft, Angela; Colombini, Manuela; *In:* BMC Medicine; 7/12/2017; v.15. 1-3. 3p. (letter) ISSN: 1741-7015 PMID: 28697810

The damage to health caused by **intimate partner violence** demands effective responses from healthcare providers and healthcare systems worldwide. To date, most evidence for the few existing, effec...

PDF Full Text PlumX Metrics

6. [Group-based microfinance for collective empowerment: a systematic review of health impacts.]

(includes abstract) Orton, Lois; Pennington, Andy; Nayak, Shilpa; Sowden, Amanda; White, Martin; Whitehead, Margaret; Bulletin of the World Health Organization, Sep2016; 94(9): 694-704A. 12p. (Article) ISSN: 0042-9686

Objective To assess the impact on health-related outcomes, of group microfinance schemes based on collective empowerment. Methods We searched the databases Social Sciences Citation Index, Embase,...

Subjects: Professional Organizations; Economics; Empowerment

Show all 5 images

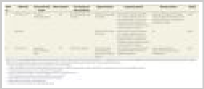

PDF Full Text PlumX Metrics

7. A nurse-delivered, clinic-based intervention to address **intimate partner violence** among **low-income** women in Mexico City: findings from a cluster randomized controlled trial.

(includes abstract) Gupta, Jhumka; Falb, Kathryn L.; Ponta, Oriana; Ziming Xuan; Abril Campos, Paola; Arellano Gomez, Annabel; Valades, Jimena; Cariño, Gisele; Diaz Olavarrieta, Claudia; Xuan, Ziming; Campos, Paola Abril; Gomez, Annabel Arellano; Olavarrieta, Claudia Diaz; BMC Medicine, 7/12/2017; 15 1-12. 12p. (journal article) ISSN: 1741-7015 PMID: 28697769

Background: Rigorous evaluations of health sector interventions addressing **intimate partner violence** (IPV) in **low**- and **middle-income countries** are lacking. We aimed to assess whether an enhanced ...

Show all 6 images

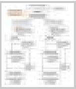

PDF Full Text PlumX Metrics

8. Association between **intimate partner violence** and poor child growth: results from 42 demographic and health surveys.

(includes abstract) Chai, Jeanne; Fink, Günther; Kaaya, Sylvia; Danaei, Goodarz; Fawzi, Wafaie; Ezzati, Majid; Lienert, Jeffrey; Smith Fawzi, Mary C.; Bulletin of the World Health Organization, May2016; 94(5): 331-339. 9p. (Article) ISSN: 0042-9686

Objective To determine the impact of **intimate partner violence** against women on children's growth and nutritional status in **low**- and **middle-income countries**. Methods We pooled records from 42 dem...

Subjects: Intimate Partner Violence; Growth In Infancy and Childhood; Child Development; Nutritional Status In Infancy and Childhood; Child: 6-12 years; Adolescent: 13-18 years; Adult: 19-44 years; **Middle** Aged: 45-64 years; Infant: 1-23 months; Child, Preschool: 2-5 years; Female; Male

Show all 5 images

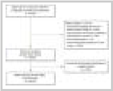

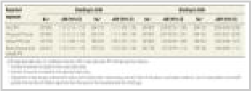

PDF Full Text PlumX Metrics

9. Preventing gender-based **violence** victimization in adolescent girls in lower-**income countries**: Systematic review of reviews.

(includes abstract) Yount, Kathryn M.; Krause, Kathleen H.; Miedema, Stephanie S.; Social Science & Medicine, Nov2017; 192 1-13. 13p. (Article) ISSN: 0277-9536

This systematic review of reviews synthesizes evidence on the impact of interventions to prevent **violence** against adolescent girls and young women 10–24 years (VAWG) in **low**- and **middle**-**income countries**...

Subjects: Domestic Violence Prevention and Control; Women's Health; **Intimate Partner Violence** Prevention and Control; Empowerment; Child: 6-12 years; Adolescent: 13-18 years; Female

Linked Full Text PlumX Metrics

10. WOMEN'S EMPOWERMENT, HOUSEHOLD STATUS AND CONTRACEPTION USE IN GHANA.

(includes abstract) Blackstone, Sarah R.; Journal of Biosocial Science, Jul2017; 49(4): 423-434. 12p. (Article) ISSN: 0021-9320 PMID: 27510983

Gender inequality is often cited as a barrier to improving women's sexual and reproductive health outcomes, including contraceptive use, in **low**- and **middle**-**income countries** such as those in **sub**-**Saharan**...

Linked Full Text PlumX Metrics

11. **Intimate Partner Violence** Reported by Disadvantaged Male Youth Varies Across **Countries**.

Rosenberg, J.; International Perspectives on Sexual & Reproductive Health, Dec2016; 42(4): 228-228. 2p. (Article) ISSN: 1944-0391

Subjects: Intimate Partner Violence Psychosocial Factors; Socioeconomic Factors; Minority Groups; Adolescent: 13-18 years

PDF Full Text PlumX Metrics

12. Attitudes Toward **Partner Violence** and Gender Roles in Uruguayan Women.

(includes abstract) Bucheli, Marisa; Rossi, Maximo; Journal of Interpersonal **Violence**, 12/1/2017; 32(23): 3693-3705. 13p. (Article) ISSN: 0886-2605

The incidence of **intimate partner violence** (IPV) in the Latin America and Caribbean region is relatively high compared with other high-**income** and **middle**-**income countries**. This problem is particul...

Subjects: Intimate Partner Violence Psychosocial Factors; Gender Role; Women Psychosocial Factors; Attitude Evaluation; Adult: 19-44 years; Female

Linked Full Text PlumX Metrics

13. [A systematic review and narrative report of the relationship between infertility, subfertility, and **intimate partner violence**.]

(includes abstract) Stellar, Carmen; Garcia-Moreno, Claudia; Temmerman, Marleen; van der Poel, Sheryl; International Journal of Gynecology & Obstetrics, Apr2016; 133(1): 3-8. 6p. (journal article) ISSN: 0020-7292 PMID: 26797197

Background: Infertility/subfertility could be a formerly unrecognized risk factor for **intimate partner violence** (IPV).Objectives: To review the evidence on the association between infertility/sub...

Subjects: Infertility Epidemiology; Study Design; Male; Female

Linked Full Text PlumX Metrics

14. A systematic review of behavioral interventions to prevent HIV infection and transmission among heterosexual, adult men in **low**-and **middle-income countries**.

(includes abstract) Townsend L; Mathews C; Zembe Y; Townsend, Loraine; Mathews, Catherine; Zembe, Yanga; Prevention Science, Feb2013; 14(1): 88-105. 18p. (journal article - research, systematic review) ISSN: 1389-4986 PMID: 23111548

Prevention of new HIV infections needs to move to the forefront in the fight against HIV and AIDS. In the current economic crisis, **low**- and **middle-income countries** (LMICs) should invest limited r...

Subjects: Behavior Therapy Methods; **Developing Countries**; Heterosexuality; HIV Infections Prevention and Control; HIV Infections Transmission; Poverty; Adolescent: 13-18 years; Adult: 19-44 years; Male

Linked Full Text PlumX Metrics

15. Setting research priorities for adolescent sexual and reproductive health in **low-** and **middle-income countries**.

(includes abstract) Hindin, Michelle J.; Christiansen, Charlotte Sigurdson; Ferguson, B. Jane; Bulletin of the World Health Organization, Jan2013; 91(1): 10-18. 9p. (Journal Article - research, tables/charts) ISSN: 0042-9686 PMID: 23397346

Objective To conduct an expert-led process for identifying research priorities in adolescent sexual and reproductive health in **low**- and **middle-income countries**. Methods The authors modified the p...

Subjects: Sexual Health Evaluation; Reproductive Health Evaluation; Research, Medical Trends; Adolescent: 13-18 years; Male; Female

Show all 5 images

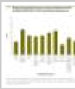
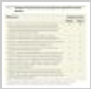
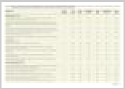
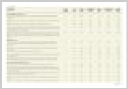

PDF Full Text PlumX Metrics

16. [Reducing child abuse amongst adolescents in **low-** and **middle-income countries**: A pre-post trial in **South** Africa.]

(includes abstract) Cluver, Lucie; Meinck, Franziska; Yakubovich, Alexa; Doubt, Jenny; Redfern, Alice; Ward, Catherine; Salah, Nasteha; De Stone, Sachin; Petersen, Tshiamo; Mpimpilashe, Phelisa; Romero, Rocio Herrero; Ncobo, Lulu; Lachman, Jamie; Tsoanyane, Sibongile; Shenderovich, Yulia; Loening, Heidi; Byrne, Jasmina; Sherr, Lorraine; Kaplan, Lauren; Gardner, Frances; BMC Public Health, 7/13/2016; 16(1): 567-567. 1p. (journal article) ISSN: 1471-2458 PMID: 27919242

Background: No known studies have tested the effectiveness of child abuse prevention programmes for adolescents in **low**- or **middle**-**income countries**. 'Parenting for Lifelong Health' (http://tiny.c...

Subjects: Parents Psychosocial Factors; Child Abuse Prevention and Control; **Developing Countries**; Parenting; Mental Health; Caregivers Psychosocial Factors; Child: 6-12 years; Adolescent: 13-18 years; Female; Male

Linked Full Text PlumX Metrics

17. Risk Factors of Physical and Sexual Abuse for Women in Mali: Findings From a Nationally Representative Sample.

(includes abstract) Hayes, Brittany E.; van Baak, Carlijn; **Violence** Against Women, Oct2017; 23(11): 1361-1381. 21p. (Article) ISSN: 1077-8012

Mali is a **developing country** that has marked inequalities between genders. Using the 2012-2013 Mali Demographic and Health Survey, a nationally representative sample of Malian women (N = 2,527), ...

Subjects: Women Psychosocial Factors; Sexual Abuse Risk Factors; **Intimate Partner Violence** Risk Factors; Socioeconomic Factors; Culture; Spouses Psychosocial Factors; Alcohol Abuse; Behavior; Adolescent: 13-18 years; Adult: 19-44 years; **Middle** Aged: 45-64 years; Female

Linked Full Text PlumX Metrics

18. **Intimate partner violence** and utilization of maternal health care services in Addis Ababa, Ethiopia.

(includes abstract) Mohammed, Bedru Hussen; Johnston, Janice Mary; Harwell, Joseph I.; Huso Yi; Katrina Wai-kay Tsang; Haidar, Jemal Ali; Yi, Huso; Tsang, Katrina Wai-Kay; BMC Health Services Research, 3/7/2017; 17 1-10. 10p. (journal article) ISSN: 1472-6963 PMID: 28270137

Background: Despite its prominence, **intimate partner violence** (IPV) against women has received little attention in Ethiopia. And as many of **sub-Saharan African countries**, maternal health care ser...

Subjects: Maternal Health Services Utilization; Patient Attitudes; Infant: 1-23 months; Adult: 19-44 years; Female

Show all 4 images

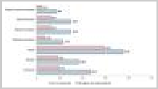
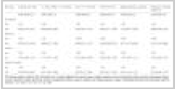

PDF Full Text PlumX Metrics

19. [Determinants of **intimate partner violence** during pregnancy among married women in Abay Chomen district, Western Ethiopia: a community based cross sectional study.]

(includes abstract) Abate, Bedilu Abebe; Wossen, Bitiya Admassu; Degfie, Tizta Tilahun; Abebe Abate, Bedilu; Admassu Wossen, Bitiya; Tilahun Degfie, Tizta; BMC Women's Health, 3/10/2016; 16 1-8. 8p. (journal article) ISSN: 1472-6874 PMID: 26960962 PMCID: PMC4785641

Background: **Intimate partner violence** during pregnancy is the most common form of **violence** that harms the health of women and the fetus but practiced commonly in **developing countries**. There is sc...

Subjects: Epidemiology; Family Conflict; Interpersonal Relations; Female

Show all 7 images

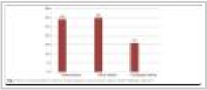

PDF Full Text PlumX Metrics

20. **Intimate partner violence** as a factor associated with risky sexual behaviours and alcohol misuse amongst men in **South** Africa.

(includes abstract) Mthembu, J. C.; Khan, G.; Mabaso, M. L. H.; Simbayi, L. C.; AIDS Care, Sep2016; 28(9): 1132-1137. 6p. (Article) ISSN: 0954-0121

Globally **intimate partner violence** (IPV) is a public health problem that can be perpetrated by both males and females, although males are more likely to inflict severe IPV-related injuries on the...

Subjects: Intimate Partner Violence South Africa; Unsafe Sex **South** Africa; Alcohol Abuse Risk Factors; Adult: 19-44 years; **Middle** Aged: 45-64 years; Male

Linked Full Text PlumX Metrics

21. Suicidality and associated risk factors in outpatients attending a general medical facility in rural Kenya.

(includes abstract) Ongeri, L.; Mcculloch, C.E.; Neylan, T.C.; Bukusi, E.; Macfarlane, S.B.; Othieno, C.; Ngugi, A.K.; Meffert, S.M.; Journal of Affective Disorders, Jan2018; 225 413-421. 9p. (journal article) ISSN: 0165-0327 PMID: 28850856

Background: **Low**-and-**Middle**-**Income**-**Countries** (LMICs) account for 75% of global suicides. While primary care populations in high-**income countries** (HIC) typically have higher prevalence of suicidal ...

Linked Full Text PlumX Metrics

22. Patterns of separation anxiety symptoms amongst pregnant women in conflict-affected Timor-Leste: Associations with traumatic loss, family conflict, and **intimate partner violence**.

(includes abstract) Silove, D.M.; Tay, A.K.; Tol, W.A.; Tam, N.; dos Reis, N.; da Costa, Z.; Soares, C.; Rees, S.; Journal of Affective Disorders, Nov2016; 205 292-300. 9p. (journal article) ISSN: 0165-0327 PMID: 27552593

Background: Adult separation anxiety (ASA) symptoms are prevalent amongst young women in **low** and **middle-income countries** and symptoms may be common in pregnancy. No studies have focused on defini...

Linked Full Text PlumX Metrics

23. **Intimate partner violence** and current tobacco smoking in **low-** to **middle-income countries**: Individual participant meta-analysis of 231,892 women of reproductive age.

Caleyachetty, Rishi; Echouffo-Tcheugui, Justin B.; Stephenson, Rob; Muennig, Peter; Global Public Health, May2014; 9(5): 570-578. 9p. (Journal Article - meta analysis, research, tables/charts) ISSN: 1744-1692 PMID: 24773510

Subjects: Intimate Partner Violence Risk Factors; Smoking; Women's Health; Economics; Adult: 19-44 years; Female

PDF Full Text PlumX Metrics

24. Physical **violence** and associated factors during pregnancy in Yirgalem town, **South** Ethiopia.

(includes abstract) Kassa, Zemenu Yohannes; Menale, Alemu Workineh; Current Pediatric Research, 2016; 20(1): 37-42. 6p. (Article) ISSN: 0971-9032

Physical **violence** during pregnancy is an endemic global problem, which endanger to mother and fetus. Even if **violence** of women right especially in **developing countries** like Ethiopia isn't progres...

Subjects: Intimate Partner Violence Epidemiology; **Intimate Partner Violence** Psychosocial Factors; Female

PDF Full Text

25. **Intimate partner violence**, consenting to HIV testing and HIV status among Zambian women.

(includes abstract) Nelson, Kara A.; Ferrance, Jacquelyn L.; Masho, Saba W.; International Journal of STD & AIDS, Sep2016; 27(10): 832-839. 8p. (journal article) ISSN: 0956-4624 PMID: 26185042

Sub-Saharan African countries are heavily burdened with HIV, which disproportionately affects women of reproductive age. Extant literature is inconsistent regarding the link between **intimate partner**...

Subjects: Intimate Partner Violence Psychosocial Factors; HIV Infections Transmission; HIV Infections Diagnosis; AIDS Serodiagnosis Statistics and Numerical Data; **Middle** Aged: 45-64 years; Adult: 19-44 years; Female

Linked Full Text PlumX Metrics

26. **Intimate Partner Violence** Victimization and Associated Factors among Male and Female University Students in 22 **Countries** in Africa, Asia and the Americas.

(includes abstract) Pengpid, Supa; Peltzer, Karl; **African** Journal of Reproductive Health, Mar2016; 20(1): 29-39. 11p. (Article) ISSN: 1118-4841

The study aimed at investigating the prevalence of **intimate partner violence** (IPV) and its associated factors among male and female university students in 22 **countries** in Africa, the Americas and...

Subjects: Intimate Partner Violence Risk Factors; Victims; Students, College; Adult: 19-44 years; Male; Female

27. Individual and community-level tolerance of spouse abuse and the association with the circumstances of first sex among youth from six **sub-Saharan African countries**.

(includes abstract) Speizer, I.S.; AIDS Care, Mar2012; 24(3): 291-300. 10p. (Journal Article - research, tables/charts) ISSN: 0954-0121 PMID: 21902559

Youth who engage in early and premarital sex are at risk of HIV and sexually transmitted infections. Most prevention programs ignore the mediating influence of the threat and experience of **violence**...

Subjects: Intimate Partner Violence; Social Attitudes; Adolescent: 13-18 years; Adult: 19-44 years; Female

Show all 4 images

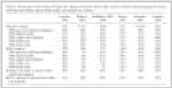
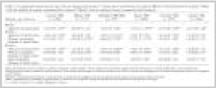
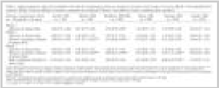

Times Cited in this Database: (1)

PDF Full Text PlumX Metrics

28. When nurses are also patients: **Intimate partner violence** and the health system as an enabler of women's health and agency in Johannesburg.

(includes abstract) Sprague, Courtenay; Woollett, Nataly; Parpart, Jane; Hatcher, Abigail M.; Sommers, Theresa; Brown, Shelley; Black, Vivian; Global Public Health, Jan/Feb2016; 11 (1/2): 169-183. 15p. (Article) ISSN: 1744-1692

While **violence** against women is a recognised global health problem, women's agency in marginalised settings is poorly understood, particularly in relation to health systems. We explored agency as...

Subjects: Nurses as Patients **South** Africa; **Intimate Partner Violence South** Africa; Decision Making; Adult: 19-44 years; **Middle** Aged: 45-64 years

Times Cited in this Database: (1)

PDF Full Text PlumX Metrics

29. [Women's approval of **domestic** physical **violence** against wives: analysis of the Ghana demographic and health survey.]

(includes abstract) Doku, David Teye; Asante, Kwaku Oppong; BMC Women's Health, 12/21/2015; 15 1-8. 8p. (journal article) ISSN: 1472-6874 PMID: 26691763 PMCID: PMC4687112

Background: **Intimate partner violence** (IPV) has serious consequences for the physical, psychological, and reproductive and sexual health of women. However, the factors that make women to justify ...

Subjects: Reproductive Health; Female

PDF Full Text PlumX Metrics

30. Keeping it in the Family: Intergenerational Transmission of **Violence** in Cebu, Philippines.

(includes abstract) Mandal, Mahua; Hindin, Michelle; Maternal & Child Health Journal, Mar2015; 19(3): 598-605. 8p. (Journal Article - research, tables/charts) ISSN: 1092-7875 PMID: 25055760

While witnessing **violence** between parents is one of the most consistent correlates of experiencing **intimate partner violence** (IPV) in later life, little research exists in **developing countries** on...

Subjects: Domestic Violence; **Intimate Partner Violence**; Family Psychosocial Factors; Child: 6-12 years; Adolescent: 13-18 years; **Middle** Aged: 45-64 years; Aged: 65+ years; Male; Female

Show all 5 images

Cited References: (34)

PDF Full Text PlumX Metrics

31. Age and **Intimate Partner Violence**: An Analysis of Global Trends Among Women Experiencing Victimization in 30 **Developing Countries.**

(includes abstract) Peterman, Amber; Bleck, Jennifer; Palermo, Tia; Journal of Adolescent Health, Dec2015; 57(6): 624-630. 7p. (Article) ISSN: 1054-139X

Purpose Young women are at elevated risk of **violence** victimization, yet generalizable evidence on age at which abuse first occurs is lacking. This analysis provides new descriptive evidence on ag...

Subjects: Violence Risk Factors; Victims; **Intimate Partner Violence**; Adult: 19-44 years; Female

Linked Full Text PlumX Metrics

32. ## In Bangla There Is No Word for Vagina.

(includes abstract) Goodman, Annekathryn; Faruque, Mithila; Clark, Rachel M.; Health (1949-4998), Sep2016; 8(12): 1244-1257. 14p. (Article) ISSN: 1949-4998

Language plays a central role in how gender and sexuality are described. In Bangla or Bengali, physicians, when educating and counseling women patients, do not have a socially acceptable word for...

Subjects: Communication Barriers Bangladesh; Social Determinants of Health; Women's Health; Sexual Health

Linked Full Text PlumX Metrics

33. ## The association between **intimate partner violence** and condom use in 36 **low**-and **middle-income countries**.

Maxwell, L; Brahmbhatt, H; Devries, K; Benedetti, A; Wagman, J; Moreno, CG; Nandi, A; Contraception, Oct2016; 94(4): 434-434. 1p. (Article) ISSN: 0010-7824

Linked Full Text PlumX Metrics

34. ## Forced Sexual Initiation, Sexual **Intimate Partner Violence** and HIV Risk in Women: A Global Review of the Literature.

Stockman, Jamila; Lucea, Marguerite; Campbell, Jacquelyn; AIDS & Behavior, Mar2013; 17(3): 832-847. 16p. (Journal Article - research, systematic review, tables/charts) ISSN: 1090-7165 PMID: 23143750

Subjects: Sexual Abuse; Coercion; **Intimate Partner Violence**; HIV Infections Risk Factors; Women's Health; Risk Taking Behavior; Adolescent: 13-18 years; Adult: 19-44 years; **Middle** Aged: 45-64 years; Aged: 65+ years; Female

Show all 8 images

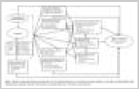
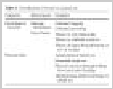
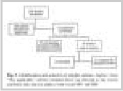

Times Cited in this Database: (5)

PDF Full Text PlumX Metrics

35. ## A systematic review of **income** generation interventions, including microfinance and vocational skills training, for HIV prevention.

(includes abstract) Kennedy, Caitlin E.; Fonner, Virginia A.; O'Reilly, Kevin R.; Sweat, Michael D.; AIDS Care, Jun2014; 26(6): 659-673. 15p. (Journal Article - research, systematic review, tables/charts) ISSN: 0954-0121 PMID: 24107189

Income generation interventions, such as microfinance or vocational skills training, address structural factors associated with HIV risk. However, the effectiveness of these interventions on HIV-...

Subjects: **Income**; HIV Infections Prevention and Control; Rehabilitation, Vocational; Prostitution Psychosocial Factors; Female

Show all 9 images

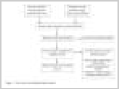

Times Cited in this Database: (1)

PDF Full Text PlumX Metrics

36. [Gender-Based **Violence** Against Adolescent and Young Adult Women in **Low**- and **Middle-Income Countries**.]

(includes abstract) Decker, Michele R.; Latimore, Amanda D.; Yasutake, Suzumi; Haviland, Miriam; Ahmed, Saifuddin; Blum, Robert W.; Sonenstein, Freya; Astone, Nan Marie; Journal of Adolescent Health, Feb2015; 56(2): 188-196. 9p. (Journal Article - meta analysis, research) ISSN: 1054-139X PMID: 25620301

Purpose Gender-based **violence** (GBV) is a global health and human rights issue with individual and social determinants. Youth are considered high risk; national influences include norms, policies ...

Subjects: Violence Epidemiology; World Health; Adolescent: 13-18 years; Female

Linked Full Text PlumX Metrics

37. Magnitude and factors associated with **intimate partner violence** in mainland Tanzania.

(includes abstract) Kazaura, Method R.; Ezekiel, Mangi J.; Chitama, Dereck; BMC Public Health, 6/10/2016; 16(1): 1-7. 7p. (journal article) ISSN: 1471-2458 PMID: 27286859 PMCID: PMC4902958

Background: In Tanzania like in many **sub-Saharan countries** the data about **Intimate Partner Violence** (IPV) are scarce and diverse. This study aims to determine the magnitude of IPV and associated ...

Subjects: Sexual **Partners** Psychosocial Factors; Adult: 19-44 years; **Middle** Aged: 45-64 years; Male; Female

Show all 6 images

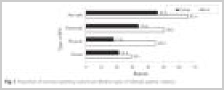

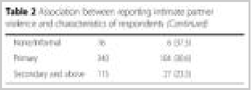

PDF Full Text PlumX Metrics

38. Psychological distress and attempted suicide in female victims of **intimate partner violence**: an illustration from the Philippines context.

(includes abstract) Antai, Diddy; Anthony, David; Journal of Public Mental Health, 2014; 13(4): 197-210. 14p. (Journal Article - research, tables/charts) ISSN: 1746-5729

Purpose - The purpose of this paper is to assess the prevalence of, and determined the factors associated with self-reported symptoms of suicide attempts and psychosocial distress among female vi...

Subjects: Intimate Partner Violence Complications; Stress, Psychological Epidemiology; Suicide, Attempted Epidemiology; Women's Health; **Intimate Partner Violence** Psychosocial Factors; Adolescent: 13-18 years; Adult: 19-44 years; **Middle** Aged: 45-64 years; Female

Linked Full Text PlumX Metrics

39. [Implementing Evidence-Based Mental Health Care in **Low-** Resource Settings: A Focus on Safety Planning Procedures.]

(includes abstract) Murray, Laura K.; Skavenski, Stephanie; Bass, Judith; Wilcox, Holly; Bolton, Paul; Imasiku, Mwiya; Mayeya, John; Journal of Cognitive Psychotherapy, 2014; 28(3): 168-185. 18p. (Journal Article - case study, tables/charts) ISSN: 0889-8391

Despite advances in global mental health evidence and policy recommendations, the uptake of evidence-based practices (EBP) in **low**- and **middle**-**income countries** has been slow. Lower resource settin...

Subjects: Professional Practice, Evidence-Based; Mental Health Services Administration; Patient Safety; Suicide Prevention and Control; Risk Management; Adult: 19-44 years; Child: 6-12 years; Adolescent: 13-18 years; Female; Male

Linked Full Text PlumX Metrics

40. Alcohol and Other Drug Use, **Partner Violence**, and Mental Health Problems Among Female Sex Workers in Southwest China.

(includes abstract) Zhang, Chen; Li, Xiaoming; Chen, Yiyun; Hong, Yan; Shan, Qiao; Liu, Wei; Zhou, Yuejiao; Health Care for Women International, Jan2014; 35(1): 60-73. 14p. (Journal Article - research, tables/charts) ISSN: 0739-9332 PMID: 23631650

In this study we investigated the association between mental health problems and negative experiences among female sex workers (FSWs) in China. A total of 1,022 FSWs completed a self-administered...

Subjects: Prostitution China; Substance Use Disorders; **Intimate Partner Violence**; Mental Disorders; Chinese Psychosocial Factors; Adolescent: 13-18 years; Adult: 19-44 years; Female

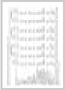 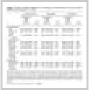 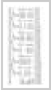

Cited References: (35) Times Cited in this Database: (1)

PDF Full Text PlumX Metrics

41. Social Factors Determining the Experience of Blindness among Pregnant Women in **Developing Countries**: The Case of India.

(includes abstract) Pandey, Shanta; Lin, Yuan; Collier-Tenison, Shannon; Bodden, Jamie; Health & Social Work, Aug2012; 37(3): 157-169. 13p. (Journal Article - research, tables/charts) ISSN: 0360-7283 PMID: 23193731

Approximately 10 million pregnant women around the world develop night blindness annually. In India, one in 11 pregnant women suffers from night blindness. This study used a nationally representa...

Subjects: Blindness In Pregnancy; Adolescent: 13-18 years; Adult: 19-44 years; **Middle** Aged: 45-64 years; Female

Cited References: (55)

PDF Full Text

42. Exploring the potential of a family-based prevention intervention to reduce alcohol use and **violence** within HIV-affected families in Rwanda.

(includes abstract) Chaudhury, Sumona; Brown, Felicity L.; Kirk, Catherine M.; Mukunzi, Sylvere; Nyirandagijimana, Beatha; Mukandanga, Josee; Ukundineza, Christian; Godfrey, Kalisa; Ng, Lauren C.; Brennan, Robert T.; Betancourt, Theresa S.; AIDS Care, 2016 Supplement; 28 118-129. 12p. (Article - research, tables/charts, randomized controlled trial) ISSN: 0954-0121

HIV-affected families report higher rates of harmful alcohol use, **intimate partner violence** (IPV) and family conflict, which can have detrimental effects on children. Few evidence-based intervent...

Subjects: Alcohol Drinking; **Domestic Violence** Prevention and Control; HIV Infections; Family Health; Social Work Service Methods; Alcohol Abuse Prevention and Control; Adult: 19-44 years; Child: 6-12 years; Adolescent: 13-18 years; **Middle** Aged: 45-64 years; Male; Female

Linked Full Text PlumX Metrics

43. Exploring the Paradox of **Intimate Partner Violence** and Increased Contraceptive Use in **sub-Saharan** Africa.

(includes abstract) Adjiwanou, Vissého; N'Bouke, Afiwa; Studies in Family Planning, Jun2015; 46(2): 127-142. 16p. (journal article) ISSN: 0039-3665 PMID: 26059986

We question the positive effect of **intimate partner violence** on women's modern contraceptive use in **sub**-**Saharan** Africa found in previous studies. The explanations offered for this counter-intuiti...

Linked Full Text PlumX Metrics

44. Is Routine Screening for **Intimate Partner Violence** Feasible in Public Health Care Settings in Kenya?

(includes abstract) Undie, Chi-Chi; Maternowska, M. Catherine; Mak'anyengo, Margaret; Askew, Ian; Journal of Interpersonal **Violence**, Jan2016; 31(2): 282-301. 20p. (Article) ISSN: 0886-2605

More than a third of women worldwide have experienced either physical and/or sexual **intimate partner violence** (IPV) or non-**partner** sexual **violence**. The short- and long-term health effects of **violence**...

Cited References: (15)

Linked Full Text PlumX Metrics

45. Is Routine Screening for **Intimate Partner Violence** Feasible in Public Health Care Settings in Kenya?

(includes abstract) Undie, Chi-Chi; Maternowska, M. Catherine; Mak'anyengo, Margaret; Askew, Ian; Journal of Interpersonal **Violence**, Jan2016; 31(1): 282-301. 20p. (Article) ISSN: 0886-2605

More than a third of women worldwide have experienced either physical and/or sexual **intimate partner violence** (IPV) or non-**partner** sexual **violence**. The short- and long-term health effects of **violence**...

Cited References: (15)

Linked Full Text PlumX Metrics

46. Women's Empowerment and Ideal Family Size: An Examination of DHS Empowerment Measures In **Sub-Saharan** Africa.

(includes abstract) Upadhyay, Ushma D.; Karasek, Deborah; International Perspectives on Sexual & Reproductive Health, Jun2012; 38(2): 78-89. 12p. (Journal Article - research, tables/charts) ISSN: 1944-0391

CONTEXT: The Demographic and Health Survey (DHS) program collects data on women's empowerment, but little is known about how these measures perform in **Sub-Saharan African countries**. It is importa...

Subjects: Women's Health; Mothers Africa **South** of the Sahara; Empowerment Evaluation; Family Relations; Adolescent: 13-18 years; Adult: 19-44 years; **Middle** Aged: 45-64 years; Female

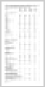 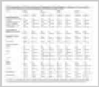 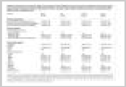

Times Cited in this Database: (2)

PDF Full Text PlumX Metrics

47. Investigating gender **violence** in Jamaica.

Spiring, Fred; **Violence** & Victims, 2014; 29(6): 1047-1076. 30p. (Journal Article) ISSN: 0886-6708 PMID: 25905144

Subjects: Developing Countries; Sexual Abuse; Perception; **Intimate Partner Violence**; Female; Male

PlumX Metrics

48. Married Women's Risk of STIs in **Developing Countries**: The Role of **Intimate Partner Violence** and **Partner**'s Infection Status.

Kishor, Sunita; **Violence** Against Women, Jul2012; 18(7): 829-853. 25p. (Journal Article - research, tables/charts) ISSN: 1077-8012

Subjects: Sexually Transmitted Diseases Risk Factors; Married Women; **Intimate Partner Violence**; Sexually Transmitted Diseases Transmission; Adolescent: 13-18 years; Adult: 19-44 years; Female; Male

Times Cited in this Database: (1)

Linked Full Text PlumX Metrics

49. Risk Factors for Antenatal Depression and Associations with Infant Birth Outcomes: Results From a **South African** Birth Cohort Study.

(includes abstract) Brittain, Kirsty; Myer, Landon; Koen, Nastassja; Koopowitz, Sheri; Donald, Kirsten A.; Barnett, Whitney; Zar, Heather J.; Stein, Dan J.; Paediatric & Perinatal Epidemiology, Nov2015; 29(6): 505-514. 10p. (journal article) ISSN: 0269-5022 PMID: 26236987

Background: Maternal antenatal depression may be particularly prevalent in **low**- and **middle-income countries**, but there is a paucity of data on its effect on birth outcomes in such settings. We in...

Subjects: Expectant Mothers Psychosocial Factors; Depression Epidemiology; Pregnancy Outcomes; Pregnancy Outcomes Psychosocial Factors; Pregnancy Complications Epidemiology; Infant, Newborn: birth-1 month; Adult: 19-44 years; Female

PDF Full Text PlumX Metrics

50. **Intimate partner violence**, modern contraceptive use and conflict in the Democratic Republic of the Congo.

(includes abstract) Kidman, Rachel; Palermo, Tia; Bertrand, Jane; Social Science & Medicine, May2015; 133 2-10. 9p. (Journal Article - research) ISSN: 0277-9536 PMID: 25828259

Intimate partner violence (IPV) has been found to be negatively associated with contraceptive use in **developing countries**, but evidence from Africa is mixed. This study examines whether the above...

Subjects: Intimate Partner Violence Epidemiology; Contraception Utilization; Female

Times Cited in this Database: (1)

Linked Full Text PlumX Metrics

Mobile Site | iPhone and Android apps | EBSCO Support Site | Privacy Policy | Terms of Use | Copyright

© 2017 EBSCO Industries, Inc. All rights reserved.
