# Supplementary material for: Are interventions focused on gender-norms effective in preventing domestic violence against women in low and lower-middle income countries? A systematic review and meta-analysis
Source: Reprod Health. 2019 Jul 1;16:93. doi: 10.1186/s12978-019-0726-5 (PMC6604322; doi:10.1186/s12978-019-0726-5)
Supplement: Supplementary file 2 — 2-1 & 2-1-1: Searching strategy on PubMed database. 2-2 Searching strategy on Medline database. 2-3 Searching strategy on EMBASE database. 2-4 Searching strategy on CNHAL database. (ZIP 2206 kb) [file 12978_2019_726_MOESM2_ESM.zip › additional file 2_1R1_1.pdf]

PubMed Advanced Search Builder

Tutorial

Use the builder below to create your search

Edit

Clear

Builder

All Fields

AND All Fields

Show index list

Show index list

Search or Add to history

History

Download history Clear history

Search	Add to builder	Query	Items found	Time
#16	Add	Search **(((domestic violence against women) OR intimate partner violence against women) AND ((associated factors OR contributing factors OR risk factors OR determinants OR predictors OR correlates OR influencing factors))) AND ((developing countries OR low and middle income countries OR poor resource setting OR least developed countries OR sub-Saharan countries OR African the south of Saharan countries OR limited resource settings OR under developed countries))**	71	06:49:38
#10	Add	Search **((((((domestic violence against women) OR intimate partner violence against women) OR physical violence against women) OR sexual violence against women) OR psychological violence against women) OR emotional violence against women) OR verbal violence against women**	3854	06:17:29
#15	Add	Search **((((((((domestic violence against women) OR intimate partner violence against women) OR physical violence against women) OR sexual violence against women) OR psychological violence against women) OR emotional violence against women) OR verbal violence against women)) AND ((developing countries OR low and middle income countries OR poor resource setting OR least developed countries OR sub-Saharan countries OR African the south of Saharan countries OR limited resource settings OR under developed countries))**	292	06:16:59
#14	Add	Search **(((((((((domestic violence against women) OR intimate partner violence against women) OR physical violence against**	39	06:15:15

Search	Add to builder	Query	Items found	Time
		**women) OR sexual violence against women) OR psychological violence against women) OR emotional violence against women) OR verbal violence against women)) AND ((women aged 15 to 49 years OR women in the reproductive age OR childbearing aged women))) AND ((developing countries OR low and middle income countries OR poor resource setting OR least developed countries OR sub-Saharan countries OR African the south of Saharan countries OR limited resource settings OR under developed countries))**		
#13	Add	Search **((((((((((domestic violence against women) OR intimate partner violence against women) OR physical violence against women) OR sexual violence against women) OR psychological violence against women) OR emotional violence against women) OR verbal violence against women)) AND ((women aged 15 to 49 years OR women in the reproductive age OR childbearing aged women))) AND ((associated factors OR contributing factors OR risk factors OR determinants OR predictors OR correlates OR influencing factors))) AND ((developing countries OR low and middle income countries OR poor resource setting OR least developed countries OR sub-Saharan countries OR African the south of Saharan countries OR limited resource settings OR under developed countries))**	19	06:14:01
#12	Add	Search **(associated factors OR contributing factors OR risk factors OR determinants OR predictors OR correlates OR influencing factors)**	1935388	06:13:04
#11	Add	Search **((((((((((domestic violence against women) OR intimate partner violence against women) OR physical violence against women) OR sexual violence against women) OR psychological violence against women) OR emotional violence against women) OR verbal violence against women)) AND ((women aged 15 to 49 years OR women in the reproductive age OR childbearing aged women))) AND (associated factors OR contributing factors OR risk factors OR determinants OR predictors OR correlates OR influencing factors)) AND ((developing countries OR low and middle income countries OR poor resource setting OR least developed countries OR sub-Saharan countries OR African the south of Saharan countries OR limited resource settings OR under developed countries))**	19	06:11:27
#9	Add	Search **(developing countries OR low and middle income countries OR poor resource setting OR least developed countries OR sub-Saharan countries OR African the south of Saharan countries OR limited resource settings OR under developed countries)**	143486	06:08:08
#8	Add	Search **(women aged 15 to 49 years OR women in the reproductive age OR childbearing aged women)**	82103	06:07:34

Search	Add to builder	Query	Items found	Time
#7	Add	Search **verbal violence against**	325	06:06:11
#6	Add	Search **emotional violence against women**	427	06:05:49
#5	Add	Search **psychological violence against women**	739	06:05:29
#4	Add	Search **sexual violence against women**	2075	06:05:08
#3	Add	Search **physical violence against women**	1207	06:04:29
#2	Add	Search **intimate partner violence against women**	1986	06:03:58
#1	Add	Search **domestic violence against women**	2514	06:03:28
