# Supplementary material for: Are interventions focused on gender-norms effective in preventing domestic violence against women in low and lower-middle income countries? A systematic review and meta-analysis
Source: Reprod Health. 2019 Jul 1;16:93. doi: 10.1186/s12978-019-0726-5 (PMC6604322; doi:10.1186/s12978-019-0726-5)
Supplement: Supplementary file 2 — 2-1 & 2-1-1: Searching strategy on PubMed database. 2-2 Searching strategy on Medline database. 2-3 Searching strategy on EMBASE database. 2-4 Searching strategy on CNHAL database. (ZIP 2206 kb) [file 12978_2019_726_MOESM2_ESM.zip › Additional file 2_ 3R1.pdf]

1 Resource selected | Hide | Change
Embase 1947 to present

Enter keyword or phrase (* or $ for truncation)
(•) **Keyword** ( ) Author ( ) Title ( ) Journal
Search

▾ **Limits** *(close)* ☐ Include Multimedia ☑ Map Term to Subject Heading

☐ Full Text ☐ Latest Update ☐ Abstracts
☐ Human ☐ Cochrane Library ☐ English Language

Publication Year - | -

Status: -, Article-in-Press Status, Conference Abstract Status, Embase Status, In-Process Status
NLM Status: -, In-Data-Review, In-Process, PubMed-not-MEDLINE, PubMed/MEDLINE, Publisher

Additional Limits | Edit Limits

Search History (25) View Saved

# ▲	Searches	Results	Type	Actions	Annotations
1	domestic violence.mp.	10742	Advanced	Display Results | More	
2	limit 1 to (full text and human and cochrane library and english language and embase status and yr="1994 -Current")	0	Advanced	Save | More	
3	intimate partner violence.mp.	5827	Advanced	Display Results | More	
4	physical violence.mp. or battered woman/ or physical violence/ or partner violence/ or sexual abuse/	25233	Advanced	Display Results | More	
5	sexual violence.mp. or sexual abuse/ or sexual violence/	15980	Advanced	Display Results | More	
6	psychological violence.mp.	416	Advanced	Display Results | More	
7	emotional violence.mp.	223	Advanced	Display Results | More	
8	women in the reporductive age.mp.	0	Advanced	Save | More	
9	childbearing age women.mp.	213	Advanced	Display Results | More	
10	women in the reproductive age.mp.	1151	Advanced	Display Results | More	
11	risk factor/ or associated factors.mp.	865073	Advanced	Display Results | More	
12	"social determinants of health"/ or determinants.mp.	165193	Advanced	Display Results | More	
13	predictors.mp. or predictor variable/	288117	Advanced	Display Results | More	
14	developing countries.mp. or developing country/	121312	Advanced	Display Results | More	
15	(low and middle income countries).mp. [mp=title, abstract, heading word, drug trade name, original title, device manufacturer, drug manufacturer, device trade name, keyword, floating subheading word]	10874	Advanced	Display Results | More	
16	least developed countries.mp.	228	Advanced	Display Results | More	
17	sub-saharan countries.mp.	478	Advanced	Display Results | More	
18	1 or 3 or 4 or 5 or 6 or 7	34443	Advanced	Display Results | More	
19	8 or 9 or 10	1364	Advanced	Display Results | More	
20	11 or 12 or 13	1249748	Advanced	Display Results | More	
21	14 or 15 or 16 or 17	130379	Advanced	Display Results | More	
22	18 and 19 and 20 and 21	0	Advanced	Save | More	
23	18 and 20 and 21	158	Advanced	Display Results | More	
24	18 and 21	582	Advanced	Display Results | More	
25	14 and 18	434	Advanced	Display Results | More	

Contract

Save | Remove | Combine with: AND | OR

Save All | Edit | Create RSS | View Saved

Options

To search Open Access content on Ovid, go to Basic Search.

Print Email Export + My Projects Keep Selected

All Range Clear 20 Per Page 1 Go Next >

Search Information

You searched:

18 and 21

Search terms used:

battered woman
countries
developed
developing
developing country
domestic
emotional
income
intimate
least
low
middle
partner
partner violence
physical
physical violence
psychological
sexual
sexual abuse
sexual violence
sub-saharan
violence

Search Returned:

582 text results

Sort By:

-

Customize Display

Filter By

Add to Search History

Selected Only (0)

Years

All Years

Current year

Past 3 years

Past 5 years

Specific Year Range

Subject
Author
Journal
Publication Type

My Projects

+ New Project

No projects available.

JBI EBP Tools

MANUAL BUILDER
PAMPHLET BUILDER
JOURNAL CLUB
RAPID
SUMARI
PACES
TAP
CAN-IMPLEMENT

---

1. **Global burden and epidemiology of adolescent nutrition: Issues and risk factors.**

Christian P.

Annals of Nutrition and Metabolism. Conference: 21st International Congress of Nutrition, ICN 2017. Argentina. 71 (Supplement 2) (pp 70), 2017. Date of Publication: 2017.

[Conference Abstract]

Publisher

S. Karger AG

Abstract + My Projects + Annotate

Abstract Reference
Complete Reference
Find Similar
Find Citing Articles
Check the Catalogue

---

2. **Injury patterns and health outcomes among pregnant women seeking emergency medical care in Kumasi, Ghana: Challenges and opportunities to improve care. <Types de blessures et resultats pour la sante chez les femmes enceintes ayant besoin d'une prise en charge medicale d'urgence a Kumasi au Ghana: defis et opportunites relatifs a l'amelioration des soins.>**

Osei-Ampofo M., Flynn-O'Brien K.T., Owusu-Dabo E., Otupiri E., Oduro G., Donkor P., Mock C., Ebel B.E.

African Journal of Emergency Medicine. 6 (2) (pp 87-93), 2016. Date of Publication: 01 Jun 2016.

[Article]

Publisher

African Federation for Emergency Medicine (E-mail: admin@afem.info)

Abstract + My Projects + Annotate

Abstract Reference
Complete Reference
Find Similar
Find Citing Articles
Full Text
Check the Catalogue

---

3. **Decisions only she should make.**

The Lancet

The Lancet. 390 (10105) (pp 1811), 2017. Date of Publication: 21 - 27 October 2017.

[Editorial]

Publisher

Lancet Publishing Group (E-mail: cususerv@lancet.com)

+ My Projects + Annotate

Complete Reference
Find Similar
Find Citing Articles
Check the Catalogue

---

4. **Conceptualizing pathways linking women's empowerment and prematurity in developing countries.**

Afulani P.A., Altman M., Musana J., Sudhinaraset M.

BMC Pregnancy and Childbirth. 17 (Supplement 2) (no pagination), 2017. Article Number: 338. Date of Publication: 08 Nov 2017.

[Article]

Publisher

BioMed Central Ltd. (E-mail: info@biomedcentral.com)

Abstract + My Projects + Annotate

Abstract Reference
Complete Reference
Find Similar
Find Citing Articles
Full Text
Check the Catalogue

---

5. **Antenatal depression and adversity in urban South Africa.**

Heyningen T.V., Myer L., Onah M., Tomlinson M., Field S., Honikman S.

Journal of Affective Disorders. 203 (pp 121-129), 2016. Date of Publication: 01 Oct 2016.

[Article]

Publisher

Elsevier B.V.

Abstract + My Projects + Annotate

Abstract Reference
Complete Reference
Find Similar
Find Citing Articles
Check the Catalogue

---

6. **The association between elevated blood lead levels and violent behavior during late adolescence: The South African Birth to Twenty Plus cohort.**

Nkomo P., Mathee A., Naicker N., Galpin J., Richter L.M., Norris S.A.

Environment International. 109 (pp 136-145), 2017. Date of Publication: December 2017.

[Article]

Publisher

Elsevier Ltd

Abstract + My Projects + Annotate

Abstract Reference
Complete Reference
Find Similar
Find Citing Articles
Check the Catalogue

☐ 7. **Food addiction: Prevalence, psychopathological correlates and associations with quality of life in a large sample.**

Nunes-Neto P.R., Kohler C.A., Schuch F.B., Solmi M., Quevedo J., Maes M., Murru A., Vieta E., McIntyre R.S., McElroy S.L., Gearhardt A.N., Stubbs B., Carvalho A.F.

Journal of Psychiatric Research. 96 (pp 145-152), 2018. Date of Publication: January 2018.

[Article]

Publisher
Elsevier Ltd

Abstract + My Projects + Annotate

Abstract Reference
Complete Reference

Find Similar
Find Citing Articles

Check the Catalogue

☐ 8. **"If he could speak, he would be able to point out who does those things to him": Experiences of violence and access to child protection among children with disabilities in Uganda and Malawi.**

Banks L.M., Kelly S.A., Kyegombe N., Kuper H., Devries K.

PLoS ONE. 12 (9) (no pagination), 2017. Article Number: e0183736. Date of Publication: September 2017.

[Article]

Publisher
Public Library of Science (E-mail: plos@plos.org)

Abstract + My Projects + Annotate

Abstract Reference
Complete Reference

Find Similar
Find Citing Articles

Full Text
Check the Catalogue

☐ 9. **'.. if you bring the kit home, you [can] get time and test together with your partner': Pregnant women and male partners' perceptions regarding female partner-delivered HIV self-testing in Uganda - A qualitative study.**

Matovu J.K.B., Buregyeya E., Arinaitwe J., Wanyenze R.K.

International Journal of STD and AIDS. 28 (13) (pp 1341-1347), 2017. Date of Publication: 01 Nov 2017.

[Article]

Publisher
SAGE Publications Ltd (E-mail: info@sagepub.co.uk)

Abstract + My Projects + Annotate

Abstract Reference
Complete Reference

Find Similar
Find Citing Articles

Check the Catalogue

☐ 10. **Post-trauma coping in the context of significant adversity: A qualitative study of young people living in an urban township in South Africa.**

Hiller R.M., Halligan S.L., Tomlinson M., Stewart J., Skeen S., Christie H.

BMJ Open. 7 (10) (no pagination), 2017. Article Number: e016560. Date of Publication: 01 Oct 2017.

[Article]

Publisher
BMJ Publishing Group (E-mail: subscriptions@bmjgroup.com)

Abstract + My Projects + Annotate

Abstract Reference
Complete Reference

Find Similar
Find Citing Articles

Full Text
Check the Catalogue

☐ 11. **Should community health workers offer support healthcare services to survivors of sexual violence? a systematic review.**

Gatuguta A., Katusiime B., Seeley J., Colombini M., Mwanzo I., Devries K.

BMC International Health and Human Rights. 17 (1) (no pagination), 2017. Article Number: 28. Date of Publication: 12 Oct 2017.

[Article]

Publisher
BioMed Central Ltd. (E-mail: info@biomedcentral.com)

Abstract + My Projects + Annotate

Abstract Reference
Complete Reference

Find Similar
Find Citing Articles

Full Text
Check the Catalogue

☐ 12. **Correlates of Sexual Violence Among Men Who Have Sex With Men in Tijuana, Mexico.**

Semple S.J., Stockman J.K., Goodman-Meza D., Pitpitan E.V., Strathdee S.A., Chavarin C.V., Rangel G., Torres K., Patterson T.L.

Archives of sexual behavior. 46 (4) (pp 1011-1023), 2017. Date of Publication: 01 May 2017.

[Article]

Abstract + My Projects + Annotate

Abstract Reference
Complete Reference

Find Similar
Find Citing Articles

Check the Catalogue

☐ 13. **Exploring the potential of a family-based prevention intervention to reduce alcohol use and violence within HIV-affected families in Rwanda.**

Chaudhury S., Brown F.L., Kirk C.M., Mukunzi S., Nyirandagijimana B., Mukandanga J., Ukundineza C., Godfrey K., Ng L.C., Brennan R.T., Betancourt T.S.

Abstract Reference
Complete Reference

Find Similar

AIDS Care - Psychological and Socio-Medical Aspects of AIDS/HIV. 28 (Supplement 2) (pp 118-129), 2016. Date of Publication: 26 May 2016.

[Article]

Publisher
Routledge (E-mail: info@tandf.co.uk)

Abstract + My Projects + Annotate

Find Citing Articles

Full Text
Check the Catalogue

☐ 14. **The burden of trauma at a district hospital in Malawi.**

Jaffry Z., Chokotho L.C., Harrison W.J., Mkandawire N.C.

Tropical Doctor. 47 (4) (pp 286-291), 2017. Date of Publication: 01 Oct 2017.

[Article]

Publisher
SAGE Publications Ltd (E-mail: info@sagepub.co.uk)

Abstract + My Projects + Annotate

Abstract Reference
Complete Reference

Find Similar
Find Citing Articles

Check the Catalogue

☐ 15. **International Emergency Psychiatry Challenges: Disaster Medicine, War, Human Trafficking, Displaced Persons.**

Jaung M., Jani S., Banu S., Mackey J.M.

Psychiatric Clinics of North America. 40 (3) (pp 565-574), 2017. Date of Publication: September 2017.

[Review]

Publisher
W.B. Saunders

Abstract + My Projects + Annotate

Abstract Reference
Complete Reference

Find Similar
Find Citing Articles

Check the Catalogue

☐ 16. **Attitudes towards intimate partner violence against women among women and men in 39 low- and middle-income countries.**

Tran T.D., Nguyen H., Fisher J.

PLoS ONE. 11 (11) (no pagination), 2016. Article Number: e0167438. Date of Publication: November 2016.

[Article]

Publisher
Public Library of Science (E-mail: plos@plos.org)

Abstract + My Projects + Annotate

Abstract Reference
Complete Reference

Find Similar
Find Citing Articles

Full Text
Check the Catalogue

☐ 17. **Preventing gender-based violence victimization in adolescent girls in lower-income countries: Systematic review of reviews.**

Yount K.M., Krause K.H., Miedema S.S.

Social Science and Medicine. 192 (pp 1-13), 2017. Date of Publication: November 2017.

[Review]

Publisher
Elsevier Ltd

Abstract + My Projects + Annotate

Abstract Reference
Complete Reference

Find Similar
Find Citing Articles

Check the Catalogue

☐ 18. **Perinatal suicidal ideation and behaviour: psychiatry and adversity.**

Onah M.N., Field S., Bantjes J., Honikman S.

Archives of Women's Mental Health. 20 (2) (pp 321-331), 2017. Date of Publication: 01 Apr 2017.

[Article]

Publisher
Springer-Verlag Wien (E-mail: michaela.bolli@springer.at)

Abstract + My Projects + Annotate

Abstract Reference
Complete Reference

Find Similar
Find Citing Articles

Full Text
Check the Catalogue

☐ 19. **Childhood abuse and adult-onset asthma among Peruvian women.**

Banerjee D., Gelaye B., Zhong Q.-Y., Sanchez S.E., Williams M.A.

Journal of Asthma. (pp 1-7), 2017. Date of Publication: 28 Aug 2017.

[Article In Press]

Publisher
Taylor and Francis Ltd (E-mail: healthcare.enquiries@informa.com)

Abstract + My Projects + Annotate

Abstract Reference
Complete Reference

Find Similar
Find Citing Articles

Check the Catalogue

20. **Forensic science and the right to access to justice: Testing the efficacy of self-examination intimate DNA swabs to enhance victim-centred responses to sexual violence in low-resource environments.**

Smith L.L., Wetton J.H., Lall G.K.M., Flowe H.D., Jobling M.A.

Science and Justice. 57 (5) (pp 331-335), 2017. Date of Publication: September 2017.

[Article]

Publisher
Forensic Science Society

Abstract + My Projects + Annotate

Abstract Reference
Complete Reference

Find Similar
Find Citing Articles

Check the Catalogue

All Range Clear 20 Per Page 1 Go Next >

Print Email Export + My Projects Keep Selected

English Français Italiano Deutsch 日本語 繁體中文 Español 简体中文 한국어

© 2017 Ovid Technologies, Inc. All rights reserved. OvidSP_UI03.27.01.112, SourceID 109407

About Us Contact Us Terms of Use
