# Supplementary material for: Are interventions focused on gender-norms effective in preventing domestic violence against women in low and lower-middle income countries? A systematic review and meta-analysis
Source: Reprod Health. 2019 Jul 1;16:93. doi: 10.1186/s12978-019-0726-5 (PMC6604322; doi:10.1186/s12978-019-0726-5)
Supplement: Supplementary file 2 — 2-1 & 2-1-1: Searching strategy on PubMed database. 2-2 Searching strategy on Medline database. 2-3 Searching strategy on EMBASE database. 2-4 Searching strategy on CNHAL database. (ZIP 2206 kb) [file 12978_2019_726_MOESM2_ESM.zip › Additional file 2R1_2.pdf]

1 Resource selected | Hide | Change

Ovid MEDLINE(R) Epub Ahead of Print, In-Process & Other Non-Indexed Citations, Ovid MEDLINE(R) Daily and Ovid MEDLINE(R) 1946 to Present

Enter keyword or phrase (* or $ for truncation)

◉ **Keyword** ○ Author ○ Title ○ Journal

Search

▾ **Limits** *(close)* ☐ Include Multimedia ☑ Map Term to Subject Heading

☐ Abstracts ☐ English Language ☐ Full Text
☐ Humans ☐ Core Clinical Journals (AIM) ☐ Latest Update

Publication Year - -

Additional Limits | Edit Limits

Search History (47) View Saved

# ▲	Searches	Results	Type	Actions	Annotations
1	domestic violence.mp. or Domestic Violence/	9436	Advanced	Display Results | More	
2	limit 1 to (english language and full text and humans and yr="1994 -Current")	1904	Advanced	Display Results | More	
3	Sexual Partners/ or intimate partner violence.mp. or Spouse Abuse/ or Domestic Violence/ or Battered Women/	29572	Advanced	Display Results | More	
4	limit 3 to yr="1994"	502	Advanced	Display Results | More	
5	husband violence.mp.	15	Advanced	Display Results | More	
6	home-based violence.mp.	0	Advanced	Save | More	
7	limit 6 to (english language and full text)	0	Advanced	Save | More	
8	in house.mp.	16589	Advanced	Display Results | More	
9	physical violence.mp. or Physical Abuse/	2167	Advanced	Display Results | More	
10	sexual violence.mp. or Sex Offenses/	9870	Advanced	Display Results | More	
11	psychological violence.mp.	353	Advanced	Display Results | More	
12	emotional violence.mp.	179	Advanced	Display Results | More	
13	verbal violence.mp.	138	Advanced	Display Results | More	
14	women aged 15 to 49 years.mp.	718	Advanced	Display Results | More	
15	women in the reproductive age.mp.	729	Advanced	Display Results | More	
16	childbearing aged women.mp.	65	Advanced	Display Results | More	
17	adult women.mp.	6320	Advanced	Display Results | More	
18	Risk Factors/ or associated factors.mp.	779700	Advanced	Display Results | More	
19	contributing factors.mp.	9459	Advanced	Display Results | More	
20	determinants.mp.	145575	Advanced	Display Results | More	
21	predictors.mp.	202349	Advanced	Display Results | More	
22	correlates.mp.	158485	Advanced	Display Results | More	
23	influencing factors.mp.	6499	Advanced	Display Results | More	
24	(low and middle income countries).mp. [mp=title, abstract, original title, name of substance word, subject heading word, keyword heading word, protocol supplementary concept word, rare disease supplementary concept word, unique identifier, synonyms]	10288	Advanced	Display Results | More	
25	Developing Countries/ or poor resource setting.mp.	73504	Advanced	Display Results | More	
26	least developed countries.mp.	210	Advanced	Display Results | More	
27	"Africa South of the Sahara"/ or sub-Saharan countries.mp.	10465	Advanced	Display Results | More	
28	southern Saharan countries.mp.	0	Advanced	Save | More	
29	limited resource settings.mp.	172	Advanced	Display Results | More	
30	Developing Countries/	73480	Advanced	Display Results | More	
31	under developed countries.mp.	103	Advanced	Display Results | More	
32	1 or 3 or 5	31115	Advanced	Display Results | More	
33	9 or 10 or 11 or 12 or 13	11795	Advanced	Display Results | More	
34	14 or 15 or 16 or 17	7813	Advanced	Display Results | More	

Contract

☐	35	18 or 19 or 20 or 21 or 22 or 23	1219542	Advanced	Display Results | More	
☐	36	24 or 25 or 26 or 27 or 29 or 31	86903	Advanced	Display Results | More	
☐	37	32 and 33 and 34 and 35 and 36	3	Advanced	Display Results | More	
☐	38	limit 37 to (english language and full text and humans and yr="1994 -Current")	0	Advanced	Save | More	
☐	39	1 or 3	31115	Advanced	Display Results | More	
☐	40	9 or 10 or 11	11679	Advanced	Display Results | More	
☐	41	14 or 15 or 17	7750	Advanced	Display Results | More	
☐	42	18 or 20 or 21 or 22	1206488	Advanced	Display Results | More	
☐	43	24 or 25 or 27	86602	Advanced	Display Results | More	
☐	44	33 and 34 and 39 and 42 and 43	3	Advanced	Display Results | More	
☐	45	((((((((domestic violence or intimate partner violence or husband violence) and physical violenceOR physical abuse) or sexual violence or psychological violence or emotional violence or verbal violence) and women aged 15 to 49 years) or women in the reproductive age or childbearing aged women or adult women) and associated factors) or contributing factors or risk factors or determinants or predictors or correlates or influencing factors) and low and middle income countries) or poor resource setting or developing countries or least developed countries or sub-Saharan countries or African south of the Sahara countries or limited resource settings or under developed countries).mp. [mp=title, abstract, original title, name of substance word, subject heading word, keyword heading word, protocol supplementary concept word, rare disease supplementary concept word, unique identifier, synonyms]	119459	Advanced	Display Results | More	
☐	46	limit 45 to (english language and full text and humans and yr="1994 -Current")	16004	Advanced	Display Results | More	
☐	47	limit 46 to (english language and female and ovid full text available and full text and humans and yr="2017" and medline)	33	Advanced	Display Results | More	

Save Remove Combine with: AND OR

Save All Edit Create RSS View Saved

To search Open Access content on Ovid, go to Basic Search.

© 2017 Ovid Technologies, Inc. All rights reserved. OvidSP_UI03.27.01.112, SourceID 109407
