# Supplementary material for: Are interventions focused on gender-norms effective in preventing domestic violence against women in low and lower-middle income countries? A systematic review and meta-analysis
Source: Reprod Health. 2019 Jul 1;16:93. doi: 10.1186/s12978-019-0726-5 (PMC6604322; doi:10.1186/s12978-019-0726-5)
Supplement: Supplementary file 3 — Studies quality assessment using JBI critical appraisal checklist. (DOCX 24 kb) [file 12978_2019_726_MOESM3_ESM.docx]

**JBI Critical Appraisal Checklist for Studies Reporting Prevalence Data**

| **Author, year** | **Criteria** |  |  |  |  |  |  |  |  | **Total** | **Overall appraisal decision** |
| --- | --- | --- | --- | --- | --- | --- | --- | --- | --- | --- | --- |
|  | **1** | **2** | **3** | **4** | **5** | **6** | **7** | **8** | **9** | 8/9 | **Included** |
| Sapkota et al.2016, (CS) | **Y** | **Y** | **Y** | **Y** | **Y** | **Y** | **Y** | **Y** | **N** | 8/9 | **Included** |
| Fikree F. et al, 2006, (CS) | **Y** | **Y** | **Y** | **Y** | **Y** | **Y** | **Y** | **Y** | **N** | 8/9 | **Included** |
| Semahegn et al, 2013. (CS) | **Y** | **Y** | **Y** | **Y** | **Y** | **un** | **Y** | **Y** | **Y** | 7/9 | **Included** |
| Ali et al, 2016. (CS) | **Y** | **Y** | **Y** | **N** | **Y** | **Y** | **Y** | **Y** | **N** | 8/9 | **Included** |
| Hayati et al. 2011, (Longitudinal) | **Y** | **Y** | **Y** | **Y** | **Y** | **Y** | **Y** | **Y** | **N** | 8/9 | **Included** |
| Doku and Asante, 2015. (longitudinal) | **Y** | **Y** | **Y** | **Y** | **Y** | **Y** | **Y** | **Y** | **N** | 6/9 | **Included** |
| Dhakal L et al, 2014. (CS) | **Y** | **Y** | **Y** | **N** | **Y** | **N** | **Y** | **Y** | **N** | 6/9 | **Included** |
| Sambisa W. et al, 2011 (CS) | **Y** | **Y** | **Y** | **N** | **Y** | **N** | **Y** | **Y** | **Y** | 7/9 | **Included** |
| Abebe Abate et al. 2016. (CS) | **Y** | **Y** | **N** | **Y** | **Y** | **N** | **Y** | **Y** | **N** | 7/9 | **Included** |
| Rapp et al, 2012, (CS) | **Y** | **Y** | **Y** | **Y** | **Y** | **N** | **Y** | **Y** | **N** | 7/9 | **Included** |
| Dalal K et al, 2014, (CS) | **Y** | **Y** | **Y** | **N** | **Y** | **N** | **Y** | **Y** | **N** | 7/9 | **Included** |
| Rahman M, 2015. (CS) | **Y** | **Y** | **Y** | **Y** | **Y** | **N** | **Y** | **Y** | **Y** | 7/9 | **Included** |
| Tumwesigye et al. 2012 (CS) | **Y** | **Y** | **N** | **Y** | **Y** | **N** | **Y** | **Y** | **N** | 6/9 | **Included** |
| Yigzaw T et al, 2004. (CS) | **Y** | **Y** | **Y** | **N** | **Y** | **N** | **Y** | **Y** | **N** | 6/9 | **Included** |
| Delamou et al, 2015, (CS) | **Y** | **Y** | **0** | **Y** | **Y** | **N** | **Y** | **Y** | **N** | 7/9 | **Included** |
| Kabir Z et al, 2014 (Longitudinal) | **Y** | **Y** | **Y** | **Y** | **N** | **N** | **Y** | **Y** | **N** | 6/9 | **Included** |
| Kazaura et al., 2016. (CS) | **Y** | **Y** | **Y** | **N** | **N** | **N** | **Y** | **Y** | **N** | 6/9 | **Included** |
| Kouyoumdjian et al.2013, (CH) | **Y** | **Y** | **N** | **Y** | **Y** | **N** | **Y** | **Y** | **N** | 6/9 | **Included** |
| Rahman et al. 2012, (CS) | **Y** | **Y** | **N** | **Y** | **Y** | **N** | **Y** | **Y** | **N** | 6/9 | **Included** |
| Deyessa N. et al, 2010 (CS) | **Y** | **Y** | **N** | **Y** | **Y** | **N** | **Y** | **Y** | **N** | 6/9 | **Included** |
| Karamagi et al, 2006. (CS) | **Y** | **Y** | **N** | **Y** | **Y** | **N** | **Y** | **Y** | **N** | 7/9 | **Included** |
| Das et al.2013 (CS) | **Y** | **Y** | **Y** | **Y** | **Y** | **N** | **Y** | **Y** | **N** | 7/9 | **Included** |
| Burgos-Soto J. et al, 2014. (CS) | **Y** | **Y** | **Y** | **Y** | **Y** | **N** | **Y** | **Y** | **Y** | 7/9 | **Included** |
| Yimer T. et al, 2014. (CS) | **Y** | **Y** | **Y** | **0** | **Y** | **N** | **Y** | **Y** | **N** | 7/9 | **Included** |
| Dalal K et al, 2013. (CS) | **Y** | **Y** | **Y** | **Y** | **N** | **N** | **Y** | **Y** | **Y** | 8/9 | **Included** |
| Eme T Owoaj et al, 2012, (CS) | **Y** | **Y** | **Y** | **Y** | **Y** | **N** | **Y** | **Y** | **N** | 6/9 | **Included** |
| Laisser et al. 2011. Qualitative | **Y** | **Y** | **Y** | **Y** | **Y** | **N** | **Y** | **Y** | **Y** | 7/9 | **Included** |
| Deribe K et al, 2012 (CS) | **Y** | **Y** | **N** | **Y** | **Y** | **N** | **Y** | **Y** | **N** | 7/9 | **Included** |
| Antai and Adaji, 2012. (CS) | **Y** | **Y** | **Y** | **Y** | **N** | **N** | **Y** | **Y** | **Y** | 7/9 | **Included** |
| Kapiga et al.2017 (CS) | **Y** | **Y** | **Y** | **N** | **Y** | **N** | **Y** | **Y** | **Y** | 8/9 | **Included** |
| Feseha et al.2012. (CS) | **Y** | **Y** | **Y** | **Y** | **N** | **N** | **Y** | **Y** | **N** | 7/9 | **Included** |
| Osinde et al, 2011. (CS) | **Y** | **Y** | **Y** | **Y** | **Y** | **N** | **Y** | **Y** | **Y** | 6/9 | **Included** |
| Yigzaw T et al 2010. (CS, Qualitative) | **Y** | **N** | **Y** | **N** | **Y** | **N** | **Y** | **Y** | **N** | 7/9 | **Included** |
| Uthman OA, et al, 2011. (CS) | **Y** | **Y** | **Y** | **Y** | **Y** | **N** | **Y** | **Y** | **N** | 8/9 | **Included** |
| Bamiwuye and Odimegwu, 2014 (CS) | **Y** | **Y** | **Y** | **Y** | **Y** | **N** | **Y** | **Y** | **Y** | 6/9 | **Included** |
| Abeya et al, 2012 qualitative | **Y** | **Y** | **Y** | **N** | **Y** | **N** | **Y** | **Y** | **N** | 7/9 | **Included** |
| Bazargan-Hejazia et al, 2013. (CS) | **Y** | **Y** | **Y** | **Y** | **Y** | **N** | **Y** | **Y** | **Y** | 7/9 | **Included** |
| Zacarias et al.2012 (CS) | **Y** | **Y** | **N** | **Y** | **Y** | **N** | **Y** | **Y** | **N** | 6/9 | **Included** |
| Meekers et al, 2013, (CS) | **Y** | **Y** | **Y** | **N** | **Y** | **N** | **Y** | **Y** | **N** | 8/9 | **Included** |
| Abeya et al, 2011. (CS) | **Y** | **Y** | **Y** | **Y** | **Y** | **N** | **Y** | **Y** | **Y** | 6/9 | **Included** |
| Koenig M. et al 2003. (CS) | **Y** | **Y** | **Y** | **N** | **Y** | **N** | **Y** | **Y** | **N** | 6/9 | **Included** |
| Wandera et al.2015 (CS) | **Y** | **Y** | **Y** | **N** | **Y** | **N** | **Y** | **Y** | **N** | 9/9 | **Included** |
| Deyessa N et al, 2009, (CS) | **Y** | **Y** | **Y** | **Y** | **Y** | **Y** | **Y** | **Y** | **Y** | 7/9 | **Included** |
| Valladares E et al, 2005. (CS) | **Y** | **Y** | **Y** | **N** | **Y** | **N** | **Y** | **Y** | **Y** | 8/9 | **Included** |

**NB:**

**JBI Critical Appraisal Checklist for Studies Reporting Prevalence Data**

|  | Yes(Y) | No(N) | Unclear(un) | Not applicable |
| --- | --- | --- | --- | --- |
| 1. Was the sample frame appropriate to address the target population? | □ | □ | □ | □ |
| 1. Were study participants sampled in an appropriate way? | □ | □ | □ | □ |
| 1. Was the sample size adequate? | □ | □ | □ | □ |
| 1. Were the study subjects and the setting described in detail? | □ | □ | □ | □ |
| 1. Was the data analysis conducted with sufficient coverage of the identified sample? | □ | □ | □ | □ |
| 1. Were valid methods used for the identification of the condition? | □ | □ | □ | □ |
| 1. Was the condition measured in a standard, reliable way for all participants? | □ | □ | □ | □ |
| 1. Was there appropriate statistical analysis? | □ | □ | □ | □ |
| 1. Was the response rate adequate, and if not, was the low response rate managed appropriately? | □ | □ | □ | □ |

Overall appraisal: Include □ Exclude □ Seek further info □

Comments (Including reason for exclusion)
