# Supplementary material for: Are interventions focused on gender-norms effective in preventing domestic violence against women in low and lower-middle income countries? A systematic review and meta-analysis
Source: Reprod Health. 2019 Jul 1;16:93. doi: 10.1186/s12978-019-0726-5 (PMC6604322; doi:10.1186/s12978-019-0726-5)
Supplement: Supplementary file 4 — 4-1 Heterogeneity between studies illustration using funnel plot. 4-2 Risk of bias (ROB) assessment. (ZIP 69 kb) [file 12978_2019_726_MOESM4_ESM.zip › Additional file 4R1_1.docx]

Funnel plot to examine publication bias

Lifetime VAW

Figure 2: Funnel plot of the lifetime intimate partner violence in LLMICs (n=19)

Lifetime physical VAW

Figure 3. Funnel plot of the Lifetime physical intimate partner violence against women (n=18).

Lifetime psychological VAW

Figure 4: Funnel plot of the lifetime psychological intimate partner violence against women (n=15)

Lifetime sexual

Figure 5: Funnel plot of the lifetime sexual intimate partner violence against women (n=15)

Current VAW

Figure 6: Funnel plot of the current intimate partner violence against women (n=33)

Current physical VAW

Figure 7: Funnel plot of the current physical intimate partner violence against women (n=31)

Current psychological VAW

Figure 8: Funnel plot of the current psychological intimate partner violence against women (n=20)

Current sexual VAW

Figure 9: Funnel plot of the current sexual intimate partner violence against women (n=27).

Concurrent VAW

Figure 10: Funnel plot of the pooled prevalence of concurrent intimate partner violence against women (n=11)
