# Supplementary material for: Are interventions focused on gender-norms effective in preventing domestic violence against women in low and lower-middle income countries? A systematic review and meta-analysis
Source: Reprod Health. 2019 Jul 1;16:93. doi: 10.1186/s12978-019-0726-5 (PMC6604322; doi:10.1186/s12978-019-0726-5)
Supplement: Supplementary file 4 — 4-1 Heterogeneity between studies illustration using funnel plot. 4-2 Risk of bias (ROB) assessment. (ZIP 69 kb) [file 12978_2019_726_MOESM4_ESM.zip › Additional file 4-2R1.docx]

Risk of bias software based finding


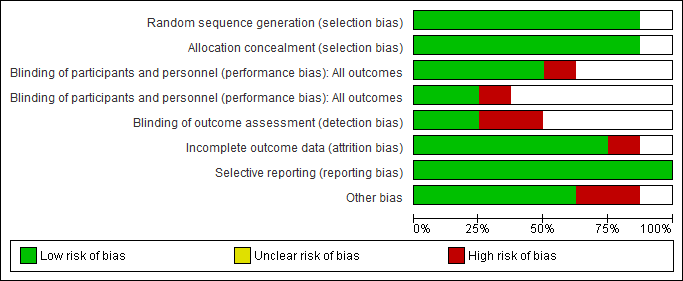


Figure: risk of bias graph


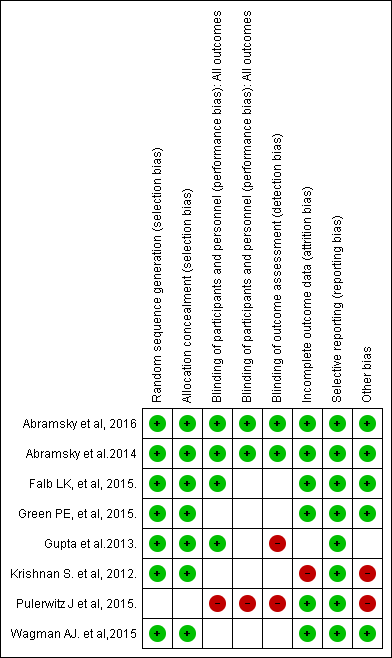
Figure: Risk of bias summary: review authors' judgments about each risk of bias item for each included study.

Table: Assessment of the risk of bias for the interventional studies (Manually)

| Studies | Radom sequence generation (selection bias) | Allocation concealment (selection bias) | Blinding of participant (Performance bias) | Blinding of assessors (detection bias) | Incomplete outcome data (Attrition bias) | Confounders ( control using: ITTA, Propensity score, others means) | Selective reporting (Reporting bias) |
| --- | --- | --- | --- | --- | --- | --- | --- |
| Abramsky et al.2014. | ⊕ | ⊕ | ⊕⊕ | ⊕⊕ | ⊕ | ⊕ | ⊕ |
| Gupta et al.2013. | ⊕ | ⊕ | ⊕⊕⊕ | ⊕⊕⊕ | ⊕ | ⊕ | ⊕ |
| Pulerwitz J et al, 2015. | ⊕⊕⊕ | ⊕⊕ | ⊕⊕⊕ | ⊕⊕⊕ | ⊕ | ⊕⊕ | ⊕ |
| Falb LK, et al, 2015. | ⊕ | ⊕ | ⊕⊕ | ⊕⊕ | ⊕ | ⊕ | ⊕ |
| Krishnan S. et al, 2012. | ⊕ | ⊕ | ⊕⊕ | ⊕⊕ | ⊕ | ⊕⊕ | ⊕ |
| Wagman AJ. et al,2015. | ⊕ | ⊕ | ⊕⊕ | ⊕⊕ | ⊕ | ⊕ | ⊕ |
| Green PE, et al, 2015. | ⊕ | ⊕ | ⊕⊕ | ⊕⊕ | ⊕ | ⊕ | ⊕ |
| Abramsky et al, 2016 | ⊕ | ⊕ | ⊕⊕ | ⊕⊕ | ⊕ | ⊕ | ⊕ |

Note that

The following four questions have to be looked in to assess the risk of bias

1. Were the baseline characteristics of the participants in the intervention and control similar?
   - Radom assignment versus selection bias

2. To what extent was the study blinded?

- - Performance bias and or Detection bias

3. Were the baseline characteristics balanced at the study completion?

- Was followed up completed (Attrition bias)?
- Was the participant analyzed to the group which was assigned /randomized
- Controlling of confounders through Per protocol analysis, Intention to treat analysis, propensity score matching and etc.

4. Were all the trials pre-specified outcome, at the pre-specified time point reported?

- - Reporting bias- time and type of reporting issues

**The rating scale of studies Risk of bias**

- High : ⊕⊕⊕⊕
- Moderate: ⊕⊕⊕
- Low : ⊕⊕
- Very Low: ⊕
