# Supplementary material for: Are interventions focused on gender-norms effective in preventing domestic violence against women in low and lower-middle income countries? A systematic review and meta-analysis
Source: Reprod Health. 2019 Jul 1;16:93. doi: 10.1186/s12978-019-0726-5 (PMC6604322; doi:10.1186/s12978-019-0726-5)
Supplement: Supplementary file 6 — GRADE for the summary of evidence for different outcomes. (DOCX 18 kb) [file 12978_2019_726_MOESM6_ESM.docx]

| **GRADE criteria for Risk of bias (ROB) and quality of evidence assessment** | | | | | | | | | |
| --- | --- | --- | --- | --- | --- | --- | --- | --- | --- |
| **Participants, Study design**  **(**RCT (starts as high quality) Non-RCT (starts as low quality)**)** | **Risk of Bias**  ( No serious (-1)  very serious (-2) | **Inconsistency**  No serious (-1)  very serious (-2) | **Indirectness**  No serious (-1)  very serious (-2)**)** | **Imprecision**  No serious (-1)  very serious (-2) | **Publication Bias (**Undetected  Strongly suspected(-1) | **Quality of the evidence** | **Study event rate (IPV/sample size )** | **Relative effect size (95% CI)** | **Remark** |
| **Outcome: Overall lifetime VAW** | | | | | | | | | |
| **35,974 ([19 observational studies]** | very serious (-2) | No serious (-1) | No serious (-1) | No serious (-1) | None | ⊕⊕ Low | 18067/35974 (50.2%) | 55% (95% CI: 52%, 59%). |  |
| **Outcome: Physical lifetime VAW** | | | | | | | | |  |
| 44,664 **(18 observational studies )** | very serious (-2) | No serious (-1) | No serious (-1) | No serious (-1) | None | ⊕⊕ Low | 19001/44664(42.5%) | 39% (95% CI: 33%, 45%) |  |
| **Outcome:** Psychological **lifetime VAW** | | | | | | | | |  |
| 42600 **( 15 observational studies )** | very serious (-2) | No serious (-1) | No serious (-1) | No serious (-1) | None | ⊕⊕ Low | 17263/ 42600(40.5%) | 46% (95% CI: 40%, 52%) |  |
| **Outcome: Sexual lifetime VAW** | | | | | | | | |  |
| **29,127 (15 observational studies )** | very serious (-2) | No serious (-1) | No serious (-1) | No serious (-1) | None | ⊕⊕ Low | 8273/29127 (31.8%) | 20% (95% CI: 17%, 23%) |  |
| **Outcome: Current VAW** | | | | | | | | |  |
| **216,043 (33 observational studies ]** | No serious (-1) | No serious (-1) | No serious (-1) | No serious (-1) | None | ⊕⊕⊕ Moderate | (98987/216043(45.8%) | 38% (95% CI: 34%, 43%) |  |
| **Outcome: Physical VAW** | | | | | | | | |  |
| **141,820 ( 31 observational studies )** | No serious (-1) | No serious (-1) | No serious (-1) | No serious (-1) | None | ⊕⊕⊕ Moderate | 34780/141820(24.5%) | 25% (95% CI: 21%, 28%) |  |
| **Outcome:** Psychological VAW | | | | | | | | |  |
| 115,798 **(20 observational studies )** | No serious (-1) | No serious (-1) | No serious (-1) | No serious (-1) | None | ⊕⊕⊕ Moderate | 27628/115798(23.9%) | 30% (95% CI: 24.0%, 36%) |  |
| **Outcome: Sexual VAW** | | | | | | | | |  |
| **124,739 (27 observational studies )** | No serious (-1) | No serious (-1) | No serious (-1) | No serious (-1) | None | ⊕⊕⊕ Moderate | 14682/124739 (11.8%) | 7% (95% CI: 7%, 8%) |  |
| **Outcome: Concurrent VAW** | | | | | | | | |  |
| **8315 (11 observational studies )** | No serious (-1) | No serious (-1) | No serious (-1) | No serious (-1) | None | ⊕⊕⊕ Moderate | 2218/8315 (26.75) | 13% (95% CI: 12%, 15%) |  |
| **Effect of intervention on VAW** | | | | | | | | |  |
| **Interventional studies (7 RCTS)** | No serious (-1) | No serious (-1) | No serious (-1) | No serious (-1) | None | ⊕⊕⊕⊕ high | Only systematic review | Only systematic review |  |
| **Interventional studies (7 RCTS)** | No serious (-1) | No serious (-1) | No serious (-1) | No serious (-1) | None | ⊕⊕⊕⊕ high | Only systematic review | Only systematic review |  |

Note that: **The rating scale of studies Quality of the evidence**

- High : ⊕⊕⊕⊕
- Moderate: ⊕⊕⊕
- Low : ⊕⊕
- Very Low: ⊕

Footnote

1. Unclear or no blinding of participants, investigators and outcome assessors and loss to follow ups ranging 0 to 56%, not downgraded for risk of bias, see imprecision
2. Few events, and wide confidence intervals, but absolute effects small in low risk group.
3. Few events, and wide confidence intervals including appreciable benefit and harm (absolute effects).
4. Unclear or no blinding of participants, investigators and outcome assessors, and loss to follow up ranging from 0 to 56%.
5. Unclear or no blinding of participants, investigators and outcome assessors, and baseline score was significantly lower in intervention group.
6. Few participants and includes potential for appreciable benefit or little to no effect
7. Unclear or no blinding of participants, investigators and outcome assessors, loss to follow up , and high risk of bias due to outcome reporting bias
