# Supplementary material for: Are interventions focused on gender-norms effective in preventing domestic violence against women in low and lower-middle income countries? A systematic review and meta-analysis
Source: Reprod Health. 2019 Jul 1;16:93. doi: 10.1186/s12978-019-0726-5 (PMC6604322; doi:10.1186/s12978-019-0726-5)
Supplement: Supplementary file 7 — Confirmation letter for funding. (PDF 1930 kb) [file 12978_2019_726_MOESM7_ESM.pdf]

UNIVERSITY OF GHANA
OFFICE OF RESEARCH INNOVATION AND DEVELOPMENT (ORID)
P. O. Box LG 571, Legon, Accra-Ghana

INTEGRI PROCEDAMUS

ORID/TDR/2016-2017/011
My Ref. No.:...

30th June 2017

TO WHOM IT MAY CONCERN

Dear Sir/Madam,

LETTER OF INTRODUCTION: AGUMASIE SEMAHEGN DEMISIE

This letter is to confirm that **Mr Agumasie Semahegn Demisie,** an Ethiopian national, has been awarded a scholarship under the **TDR International Postgraduate Training Scheme to pu**rsue a four-year PhD programme in Public Health at the University of Ghana, from **January 2016 – December 2019.**

TDR, the Special Programme for Research and Training in Tropical Diseases, has provided the award. TDR is hosted at the World Health Organization (WHO) in Geneva, Switzerland, where it is co-sponsored by UNICEF, UNDP, the World Bank and WHO.

The TDR scholarship makes provision for research activities, including publication, as part of his PhD programme.

Do find enclosed a copy of his award letter.

Thank you.

Sincerely,

Empi A. Baryeh
Assistant Registrar/TDR project administrator

cc: Pro Vice-Chancellor (RID)
TDR-UG Project Coordinator

Tel.: + 233 303930436 /+ 233 302 213850 ext. 2712/2713 | Fax: + 233 302 522860 | Email: orid@ug.edu.gh | Website: http://orid.ug.edu.gh
(Location: Ground Floor-LECIAD Building)

UNIVERSITY OF GHANA
OFFICE OF RESEARCH INNOVATION AND DEVELOPMENT (ORID)

P. O. Box LG 571,Legon, Accra-Ghana

My Ref. No. ORID/TDR/I/FX/2015-2016/013

9th November, 2015

agucell@yahoo.com; agumae21@gmail.com

Dear Mr Agumasie Semahegn Demisie,

NOTICE OF AWARD:
TDR INTERNATIONAL POSTGRADUATE TRAINING SCHEME

Congratulations! You have been selected to receive a full-time scholarship under the TDR International Postgraduate Training Scheme to undertake a FOUR-YEAR **PhD in Public Health** at the University of Ghana. The scholarship will run from **January 2016 to December 2019** – no payments will be made to you for any work conducted after this date.

The scholarship includes the following:

Tuition, Research and Learning Costs
The scholarship will cover the official **fees** for your programme, which may include tuition, registration, examination, library, laboratory consumable costs, language course, etc. This will be paid directly to the University of Ghana.

The scholarship will also cover the costs of your research project for your **thesis/dissertation.**

You will be provided with a **laptop computer** and have access to the electronic resources publications of the University of Ghana Library System.

Additionally, the scholarship will cover your participation in **PhD Short Courses** on topics relevant to Implementation Research organised by the School of Public Health.

Stipend
The scholarship includes a monthly subsistence allowance as per WHO stipend rates.

Insurance
Full insurance coverage (medical, accident) will be provided for the duration of the grant.

Travel costs
The scholarship will cover the cost of one economy class return ticket between your home country and the University of Ghana.

Visa, Vaccinations, Travel Insurance, Residence permit etc.
The scholarship will reimburse the direct cost of other travel related activities such as Visa, Vaccinations, Residence permit etc., upon submission of the original receipts.

Resettlement Allowance
A one-off supplement will be paid to you as a contribution to your costs of removal

Housing
The Office of Research, Innovation and Development will assist you to find suitable accommodation at the Graduate Hostels on campus. **You will be responsible to pay for the cost of accommodation through your stipend.**

Office space
You will be provided with a workspace in the PhD students' room at the School of Public Health.

Tel: +233 303 930436 / +233 302 213850 ext. 2712/2713 | Fax: +233 302 522860 | Email:orid@ug.edu.gh | Website:http://orid.ug.edu.gh
(Location: Ground Floor -LECIAD Building)

CONDITIONS FOR THE TDR GRANT

i. You are required to submit a progress report on your programme at the end of each semester.
ii. You are encouraged to publish at least two articles from your PhD research in an internationally recognised academic journal within two years of completion of your programme.
iii. You are required to acknowledge the support of the TDR International Postgraduate Training Scheme and the University of Ghana and in all publications/reports based on your thesis research.
iv. At the end of your programme, you will be required to submit a *Programme Completion Report* to the WHO/TDR and the Programme Secretariat at the University of Ghana.
v. Any Intellectual Property that emanates from your research shall be owned by the University of Ghana.
vi. If you withdraw from the scholarship or are unable to complete the programme for any reason, you may be required to repay some or all of the scholarship funds already received. In this event, you will be responsible to repay the grant according to an agreed schedule to be set by the WHO/TDR.

All requests for disbursement of funds should be submitted to the *Project Coordinator, TDR International Postgraduate Training Scheme*, through your Principal Supervisor, who will be expected to comment/certify the request, and should be copied to your Head of Department at the University of Ghana.

You are requested to indicate your acceptance of the terms and conditions of this grant by signing below. You shall return one copy of this letter to the Pro-Vice-Chancellor (RID) with the completed additional forms (enclosed), by close of business on **Wednesday 18th November, 2015.**

For further information, please contact the Programme Secretariat at the Office of Research, Innovation and Development (ORID), University of Ghana.

Congratulations on your award. It is my hope that you will devote time and attention to complete your study in the shortest time possible.

Yours sincerely,

[signature]

Professor John Gyapong
Pro-Vice Chancellor (RID)

cc: Vice-Chancellor
Pro Vice-Chancellor (ASA)
Director, Finance Directorate
Dean, School of Graduate Studies
Provost, College of Health Sciences
Dean, School of Public Health
Dr Olumide Ogundahunsi, TDR

**ACCEPTING OFFER:**	
Please tick the shaded box to indicate your acceptance or decline	
✓	I confirm to have carefully read, be fully aware of and accept the grant and guidelines laid down. I will ensure that I submit my thesis on schedule.
**Name:** Agumasie Semahegn Demisie	
**Signature:** [signature]	**Date:** 11th November, 2015

**DECLINING OFFER:**		
**Reason for declining:**		
**Name:**	**Signature:**	**Date:**
